# Transcriptome comparison analyses in UV-B induced AsA accumulation of *Lactuca sativa* L

**DOI:** 10.1186/s12864-023-09133-7

**Published:** 2023-02-03

**Authors:** Hua Zhou, Lei Yu, Shujuan Liu, Anfan Zhu, Yanfang Yang, Caihui Chen, Aihong Yang, Lipan Liu, Faxin Yu

**Affiliations:** 1 The Key Laboratory of Horticultural Plant Genetic and Improvement of Jiangxi Province, Institute of Biological Resources, Jiangxi Academy of Sciences, Nanchang, China; 2 College of Forestry, Jiangxi Agricultural University, Nanchang, 330045 China; 3 Jiangxi Agricultural Technology Extension Center, Nanchang, 330046 China; 4 State Key Laboratory of Tree Genetics and Breeding, Key Laboratory of Tree Breeding and Cultivation of State Forestry Administration, Research Institute of Forestry, Chinese Academy of Forestry, Beijing, 100091 China

**Keywords:** AsA, UV-B, Dose, Lettuce, Transcriptome

## Abstract

**Background:**

Lettuce (*Lactuca sativa* L.) cultivated in facilities display low vitamin C (L-ascorbic acid (AsA)) contents which require augmentation. Although UV-B irradiation increases the accumulation of AsA in crops, processes underlying the biosynthesis as well as metabolism of AsA induced by UV-B in lettuce remain unclear.

**Results:**

UV-B treatment increased the AsA content in lettuce, compared with that in the untreated control. UV-B treatment significantly increased AsA accumulation in a dose-dependent manner up until a certain dose.. Based on optimization experiments, three UV-B dose treatments, no UV-B (C), medium dose 7.2 KJ·m^− 2^·d^− 1^ (U1), and high dose 12.96 KJ·m^− 2^·d^− 1^ (U2), were selected for transcriptome sequencing (RNA-Seq) in this study. The results showed that C and U1 clustered in one category while U2 clustered in another, suggesting that the effect exerted on AsA by UV-B was dose dependent. *MIOX* gene in the myo-inositol pathway and *APX* gene in the recycling pathway in U2 were significantly different from the other two treatments, which was consistent with AsA changes seen in the three treatments, indicating that AsA accumulation caused by UV-B may be associated with these two genes in lettuce. *UVR8* and *HY5* were not significantly different expressed under UV-B irradiation, however, the genes involved in plant growth hormones and defence hormones significantly decreased and increased in U2, respectively, suggesting that high UV-B dose may regulate photomorphogenesis and response to stress via hormone regulatory pathways, although such regulation was independent of the UVR8 pathway.

**Conclusions:**

Our results demonstrated that studying the application of UV-B irradiation may enhance our understanding of the response of plant growth and AsA metabolism-related genes to UV-B stress, with particular reference to lettuce.

**Supplementary Information:**

The online version contains supplementary material available at 10.1186/s12864-023-09133-7.

## Background

Vitamin C (L-ascorbic acid (AsA)), which is a nutrient essential for human growth, reproduction, and health, performs an important antioxidant function in organisms. AsA cannot be synthesised by humans and must be obtained from food sources, especially plants. Increasing the AsA content has become an important issue in promoting the nutritional quality of plants [1, 2]. AsA also plays an important role in plants, by not only regulating defence and survival via the modification of gene expression, but also modulating plant growth via phytohormones [3].

**Fig. 1:**
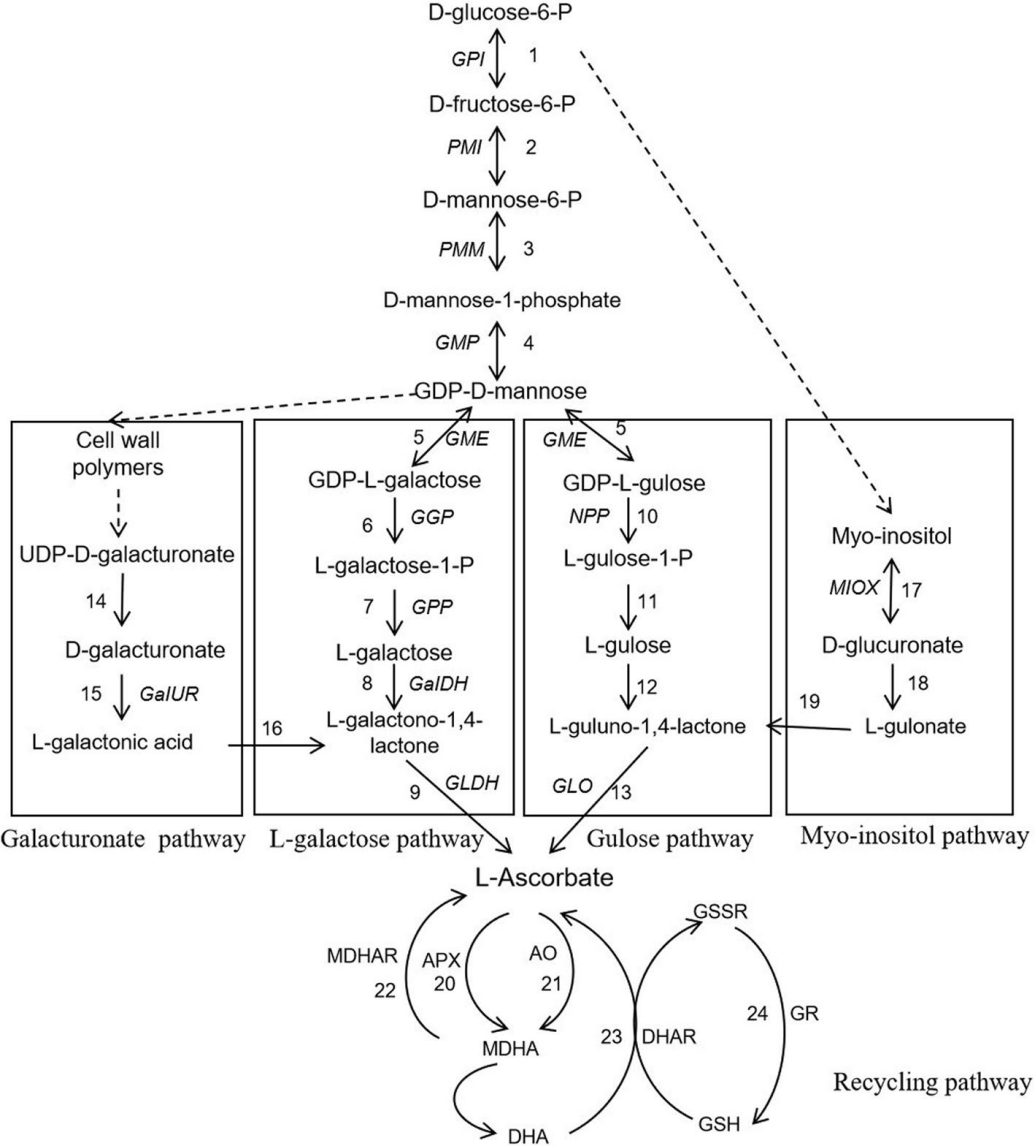
Biosynthesis and recycling pathways of ascorbic acid in plants. GPI: glucose-6-phosphate isomerase 2. PMI: mannose − 6- phosphate isomerase 3. PMM: phosphomannomutase 4. GMP: GDP- D-mannose pyrophosphorylase 5. GME:GDP-Dmannose-3′,5′-epimerase 6. GGP: GDP- L-galactose- phosphatase 7. GPP: L-galactose-1-phosphate phosphatase 8. GaIDH: L-galactose dehydrogenase 9. GLDH: L-galactono-1,4-lactone dehydrogenase 10. D-galacturonate-1-phosphate uridyltransferase 11. GaIUR: D-galacturonate reductase 12. aldonolactonase 13. NPP: nucleotide pyrophosphatase 14. sugar phosphatase 15. sugar dehydrogenase 16. GLO: L-gulono-1,4-lactone oxidase 17. MIOX: myo-inositol oxygenase 18. D-glucuronate reductase 19. L-gulonolactonase 20. APX: ascorbate peroxidase 21. AO: ascorbate oxidase 22. MDHAR: monodehydroascorbate reductase 23. DHAR: dehydroascorbate reductase 24. GR: glutathione reductase Note: Double arrows represent two-way reversible reactions, the single arrows represent one-way reactions, and the dotted arrows represent indirect reactions

Four main pathways are involved in the synthesis of AsA in higher plants; these are the L-galactose, galacturonate, glucose, and myo-inositol pathways [4, 5]. The L-galactose pathway is considered to be the major biosynthetic pathway of AsA in plants [6], and several key structural genes, including GDP- D-mannose pyrophosphorylase (*GMP*)*,* GDP-Dmannose-3′,5′-epimerase (*GME*)*,* GDP- L-galactose- phosphatase (*GGP*)*,* L-galactose dehydrogenase (*GaIDH*)*,* and L-galactono-1,4-lactone dehydrogenase (*GLDH*), which are involved in the L-galactose pathway have been isolated from some plants and identified. These genes play an important regulatory role in increasing the AsA content of plants (Fig. 1; Steps 5–9) [7]. The galacturonate pathway is an alternative pathway that contributes to AsA accumulation in strawberries (*Fragaria* × *ananassa*) (Fig. 1;Steps 10–12) and may jointly regulate AsA levels together with other pathways via *D-galacturonate reductase* (*GaIUR*); its overall effect being associated with the ripening of fruits [8]. The biosynthesis of ascorbic acid in strawberry fruits and grape (*Vitis vinifera*) berries occurs through D-galacturonic acid, and *GalUR* overexpression in *A. thaliana* may increase the vitamin C content by 2–3 times [9]. The glucose pathway is similar to that linked to AsA synthesis in animals (Fig. 1; Step 13–16). GDP-D-mannose forms GDP-L-glucose through *GME*, which is reversible in plants. GDP-L-glucose forms L-glucose-1-P via nucleotide pyrophosphatase (*NPP*), and is eventually converted to AsA [10]. The myo-inositol pathway is similar to the mammalian AsA synthesis pathway; myo-inositol generates glucuronic acid through *myo-inositol oxygenase* (*MIOX*) and, thus, synthesises AsA (Fig. 1; Steps 17–19) [11].

In plant cells, vitamin C is converted to monodehydroascorbate (MDHA) via oxidation of ascorbate peroxidase (APX) and ascorbate oxidase (AO). MDHA is reconverted to ascorbic acid via the catalytic action of monodehydroascorbate reductase (MDHAR) (Fig. 1; Steps 20–22). MDHA is converted to DHA via a non-enzymatic reaction, and subsequently DHA is reduced to AsA by dehydroascorbate reductase (DHAR). The action of DHAR depends on the coupling between glutathione reductase (GR) and glutathione (GSH) (Fig. 1; Steps 23–24) [12]. Thus, AsA recycling is evidently an important process that regulates the AsA content in plants.

Light is an important regulator of AsA levels in plants [13]. Both the quality and quantity of light influence AsA levels in plants [14]. Compared to white light, blue light, or a combination of red and blue light, increased AsA content in leaf lettuce and komatsuna (*Brassica rapa var. perviridis*) [15]. Seven days of shading led to a strong reduction in the AsA content of tomato (*Solanum lycopersicum*) leaves, which was coincide with the downregulation of *GMP, GPP, GME,* and *GGP* expression in these leaves [7]. Light increases AsA and glutathione contents by enhancing the activities of enzymes, such as MDHAR, DHAR, APX, CAT and GR, as well as by affecting the expression levels of genes, such as *GME, GDP, GPP, GaIDH,* and *GLDH*, in tomatoes [16]. *A. thaliana* leaves accumulate more AsA after acclimatization to high light intensity [17]. Light induced regulation of AsA also involves respiration and photosynthesis, which produce soluble carbohydrates that act as a signalling pathway that regulates AsA levels via stimulation of the AsA biosynthetic pathway and the recycling pathway [13].

Ultraviolet-B (UV-B), a form of high solar spectrum ultraviolet radiation with a wavelength between 280 nm and 315 nm, is considered as an abiotic stress factor that triggers reactive oxygen species (ROS) accumulation, which affects development and harms plants. To balance ROS production, plants activate a scavenging system which accumulates antioxidant substances to reduce ROS levels [18]. AsA is an important antioxidant that enhances tolerance to UV-B stress. Some ascorbate-deficient mutants of *A. thaliana* have proved to be hypersensitive to UV-B radiation [19, 20]. Moreover, most AsA increases in response to UV-B radiation are observed at high UV-B dose [21]. For example, compared with the control, AsA content in cucumber (*Cucumis sativus*) seedling leaves was significantly higher in seedlings exposed to 1.44–20.16 kJ m^− 2^ of UV-B [22]. However, UV-B does not always enhance AsA content, as this also depends on the plant species and irradiation dose [13, 21]. For instance, UV-B irradiation significantly improved AsA content in rice (*Oryza sativa*) after 14 days, after which there was a gradual decrease in AsA, with no difference between the UV-B and control plants after 28 days [21, 23].

Leaf lettuce is an important vegetable crop that is cultivated worldwide. It is popular because it is very nutritious and tastes good even when eaten raw. Protected facilities produce good quality lettuce and facilitate year-round supplies. However, lettuce cultivated in greenhouses or plant factories usually contains lower AsA levels [24]. Although, continuous LED light reportedly increases AsA levels in lettuce [25], increasing light intensity and irradiation time requires more energy. Compared to those grown under UV-blocking films in greenhouses, lettuce grown under UV-transparent films exhibits changes in plant metabolism, including that of AsA, flavonoids, and phenolics [26–28]. Therefore, regulating light quality (i.e., shorter UV-B application time) is deemed as a potential strategy that may be utilised to improve the AsA content of commercial crops grown in facilities [29, 30]. However, a detailed understanding of the effects exerted by UV-B irradiation on AsA levels in lettuce is lacking.

Therefore, we aimed to study the effects of UV-B dose on the AsA content and growth of lettuce. The transcriptome was investigated to clarify the regulatory role of UV-B dose on AsA biosynthesis in lettuce. These results are expected to provide the basic data required for a better understanding of the mechanisms underlying UV-B application induced increases in the AsA levels of lettuce grown in plant factories and facilities.

## Results

### Effect of UV-B dose on AsA content and growth in lettuce

**Fig. 2:**
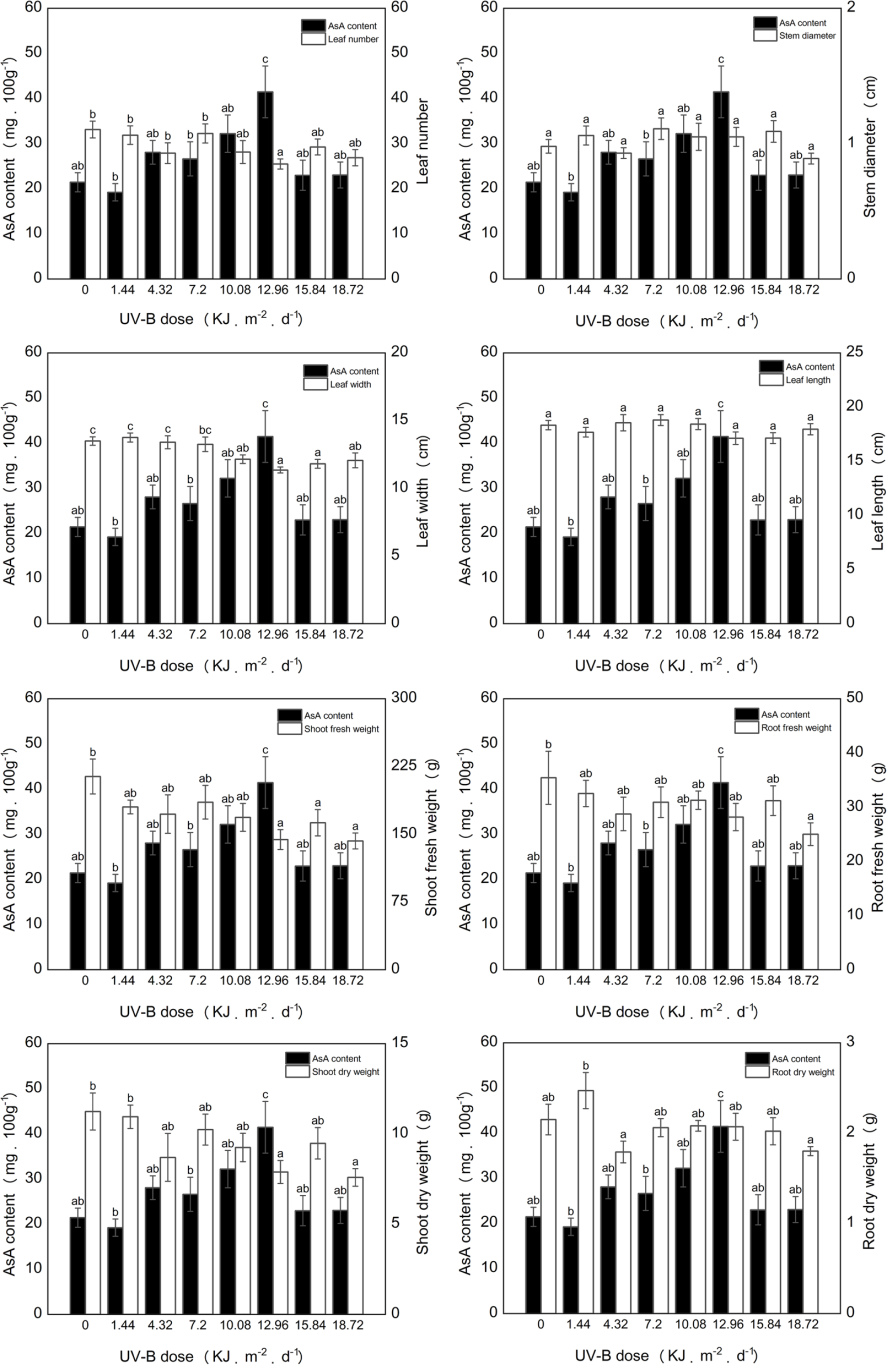
Effect of UV-B dose on ascorbic acid (AsA) in lettuce. Different letters in each histogram indicate significant difference between the treatments (*P* < 0.05). The error bars indicate the standard errors of three biological replicates

We used eight UV-B doses in a plant factory setting. Based on Brosché and Stride [31], the UV-B dose was divided into four classes: very low dose (0–1 KJ·m^− 2^·d^− 1^); low dose (1–2 KJ·m^− 2^·d^− 1^); intermediate dose (2–8 KJ·m^− 2^·d^− 1^); and high dose (8–18 KJ·m^− 2^·d^− 1^). The eight UV-B doses used in this study are listed (Fig. 2). Although there was no increase in AsA levels at the low UV-B dose (1.44 KJ·m^− 2^·d^− 1^), AsA contents increased gradually from 4.32 to 12.96 KJ·m^− 2^·d^− 1^, reaching the highest level at a UV-B dose of 12.96 KJ·m^− 2^·d^− 1^ (Fig. 2). AsA levels were significantly higher under 12.96 KJ·m-2·d-1 than that under the other treatments. However, as UV-B continued to increase and reached 15.84 and 18.72 KJ·m^− 2^·d^− 1^, the AsA content began to decrease, reaching a level that was not significantly different from that of the control.

The leaf number, shoot fresh weight, root fresh weight, and shoot dry weight of lettuce in the control treatment were higher than those of all other treatments (Fig. 2). No significant differences were observed between the stem diameters of treatments or between their leaf lengths. The leaf width of lettuce under high UV-B doses (12.96, 15.84, and 18.72 KJ·m^− 2^·d^− 1^) was significantly shorter than that under low UV-B doses (0, 1.44, and 4.32 KJ·m^− 2^·d^− 1^). Shoot fresh weight of the control was significantly higher than that of the high UV-B dose (12.96, 15.84, and 18.72 KJ·m^− 2^·d^− 1^). No significant difference was observed between the growth biomasses corresponding to UV-B doses of 0, 1.44, 4.32, and 7.20 KJ·m^− 2^·d^− 1^.

### Transcriptome analysis of lettuce under different UV-B doses

To further analyse the effects of UV-B on AsA accumulation, we performed high-throughput RNA-Seq for UV-B doses of 0 (C), 7.2 KJ·m^− 2^·d^− 1^ (U1) and 12.96 KJ·m^− 2^·d^− 1^ (U2). Since most AsA increases are observed at a high UV-B dose (8–18 KJ·m^− 2^·d^− 1^), U1 as the starting point of UV-B high dose, and U2 as a high AsA content-inducing UV-B dose were chosen for RNA-Seq in this study. U2 (high-dose) had a higher AsA content than U1 (medium dose) and C (control), with no significant difference being observed between C and U1 (Fig. 2). We obtained 84.64 Gb of clean data, with a Q30 base percentage greater than 94.82%. According to the reference genome of *Lactuca sativa* (http://lgr.genomecenter.ucdavis.edu), the percentage of uniquely mapped sequences was between 93.91 and 95.86%. Using the StringTie software (http://ccb.jhu.edu/software/stringtie/) [32], we assembled the mapped reads and obtained 60,549 unigenes, of which more than 60% were 1000 bp in length, including 38,911 (64.26%) known genes and 21,634 (35.73%) novel genes. BLAST alignment was utilised to annotate the 25,430 unigenes of lettuce with an E-value threshold of 1e^− 5^ in public databases: non-redundant proteins sequence database (Nr), annotated protein sequence database (Swiss-Prot), the protein families database (Pfam), clusters of orthologous groups of proteins (EggNOG), gene ontology (GO), and kyoto encyclopedia of genes and genomes (KEGG). The database annotation results showed that more than 99% of the genes and transcripts were successfully annotated in at least one database.

Sample correlation analysis showed that the sample repetition coefficients of C, U1, and U2 were above 0.9, with C and U1 being clustered into one category first, and U2 into another category (Additional file 1). Principal component analysis (PCA) also showed that the three treatments were clustered into three categories, with a larger distance between U2 and C than between U1 and C (Additional file 2). These results suggested that the effect of UV-B on AsA in lettuce was dose dependent.

### Analysis of differential gene expression in the lettuce transcriptome under UV-B radiation

The statistics of differentially expressed genes (DEGs) between U2, U1 and C were analysed. Compared with C, U2 carried 1402 DEGs, of which 629 were upregulated and 773 downregulated. Compared with U1, U2 carried 809 DEGs, of which 351 were upregulated genes and 458 were downregulated. Compared with C, U1 carried only 519 DEGs, of which 196 were upregulated and 323 downregulated. This suggests that, in lettuce, UV-B radiation changed mRNA transcript levels first.

**Fig. 3:**
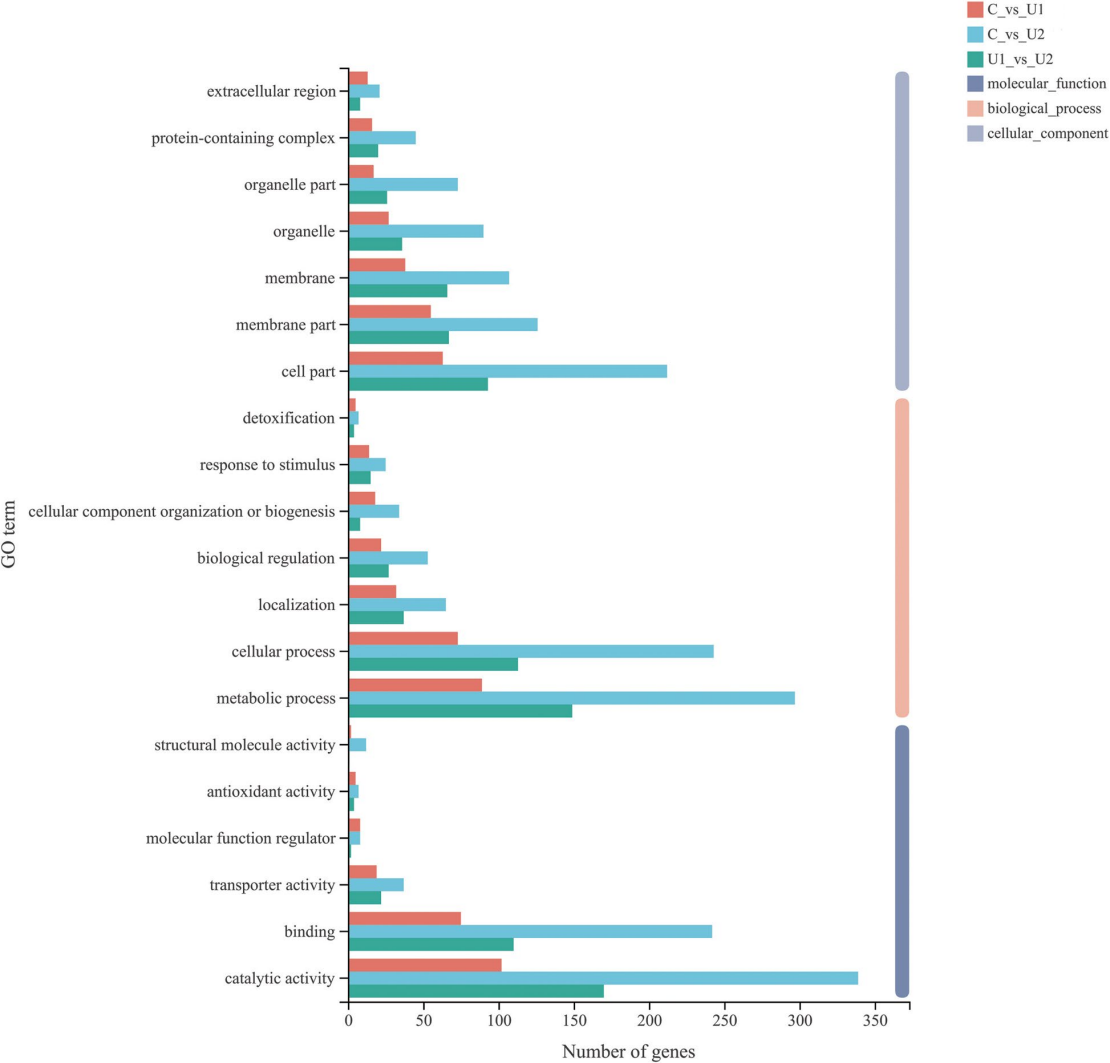
Gene ontology (GO) classification of differentially expressed genes (DEGs) in lettuce. GO terms are classified into three main categories: biological process, cellular component, and molecular function. The x-axis represents number of DEGs. The y-axis represents the enriched GO terms

GO annotation analysis was performed to explore the function of these DEGs in response to UV-B dosage, and the GO terms showed a similar distribution in the three comparisons (Fig. 3). Within the category of biological processes, DEGs were cantered on metabolic processes, cellular processes, biological regulation, and responses to stimuli, whereas catalytic activity, binding, and transporter activity were the three most abundant terms linked to molecular function. In terms of cellular components, the cell, membrane, membrane organelle, and organelle parts were most highly represented.

**Fig. 4:**
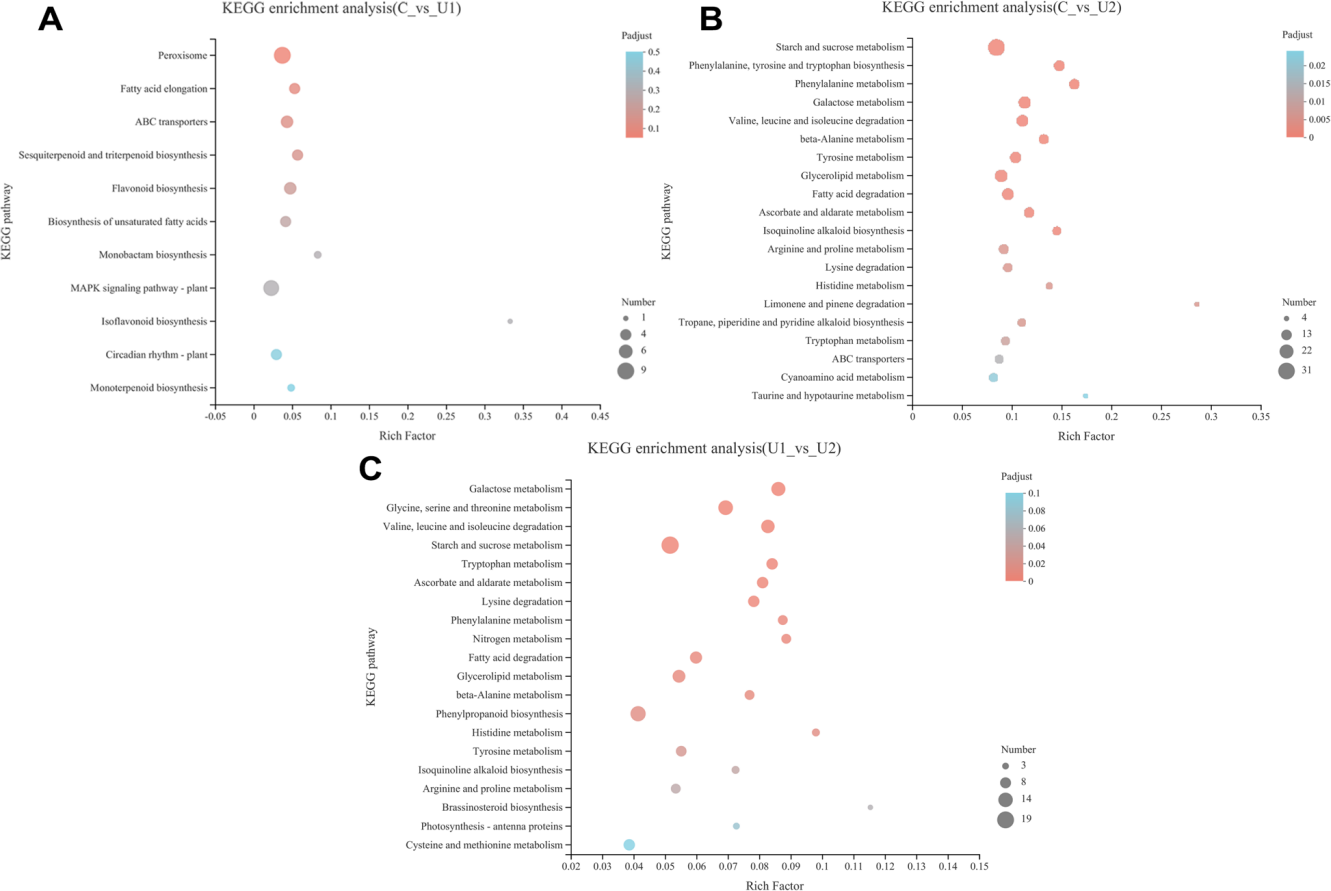
Kyoto encyclopedia of genes and genomes (KEGG) enrichment analysis of differentially expressed genes in lettuce. **A**: KEGG enrichment pathways of C and U1; **B**: KEGG enrichment pathways of C and U2; **C**: KEGG enrichment pathways of U1 and U2. The y-axis indicates the KEGG metabolic pathway. The lower x-axis indicates the number of genes annotated to the pathway. The upper x-axis represents the significance level of enrichment. The top 20 enrichment results are displayed as *P*-adjust < 0.5 by default

KEGG enrichment analysis of the DEGs was performed (Fig. 4). The results indicated that 100 KEGG pathways were enriched in U2 compared to C, where the top 20 significantly enriched pathways (*P*-adjust < 0.5) included carbohydrate metabolism (starch and sucrose metabolism, galactose metabolism, and ascorbate and aldarate metabolism), amino acid metabolism (phenylalanine, tyrosine, and tryptophan biosynthesis; phenylalanine metabolism; valine, leucine, and isoleucine degradation, and tyrosine metabolism), lipid metabolism (glycerolipid metabolism, fatty acid degradation, arachidonic acid metabolism), membrane transport (ATP binding cassette (ABC) transporters), metabolism of terpenoids and polyketides (limonene and pinene degradation, brassinosteroid biosynthesis), and other secondary metabolites (isoquinoline alkaloid biosynthesis). Similarly, when comparing U1 and U2, DEGs were associated with 99 KEGG pathways, wherein carbohydrate metabolism, amino acid metabolism, energy metabolism, lipid metabolism, and the biosynthesis of secondary metabolites were enriched (*P*-adjust < 0.5). These results indicated that ascorbate and aldarate metabolism, galactose metabolism and starch and sucrose metabolism were main key enriched pathways in U2 when compared with C and U1. The enrichment of these pathways can explain the high AsA ontent in lettuce treated with a high dose of UV-B (U2).

Compared to C, the enriched pathways in U1 included 11 pathways (*P*-adjust < 0.5), such as peroxisome, fatty acid elongation, ABC transporters, secondary metabolite biosynthesis (sesquiterpenoid, triterpenoid, flavonoid, monobactam, isoflavonoid, and monoterpenoid), mitogen-activated protein kinase (MAPK) signalling pathway - plant, and circadian rhythm-plant.

**Fig. 5:**
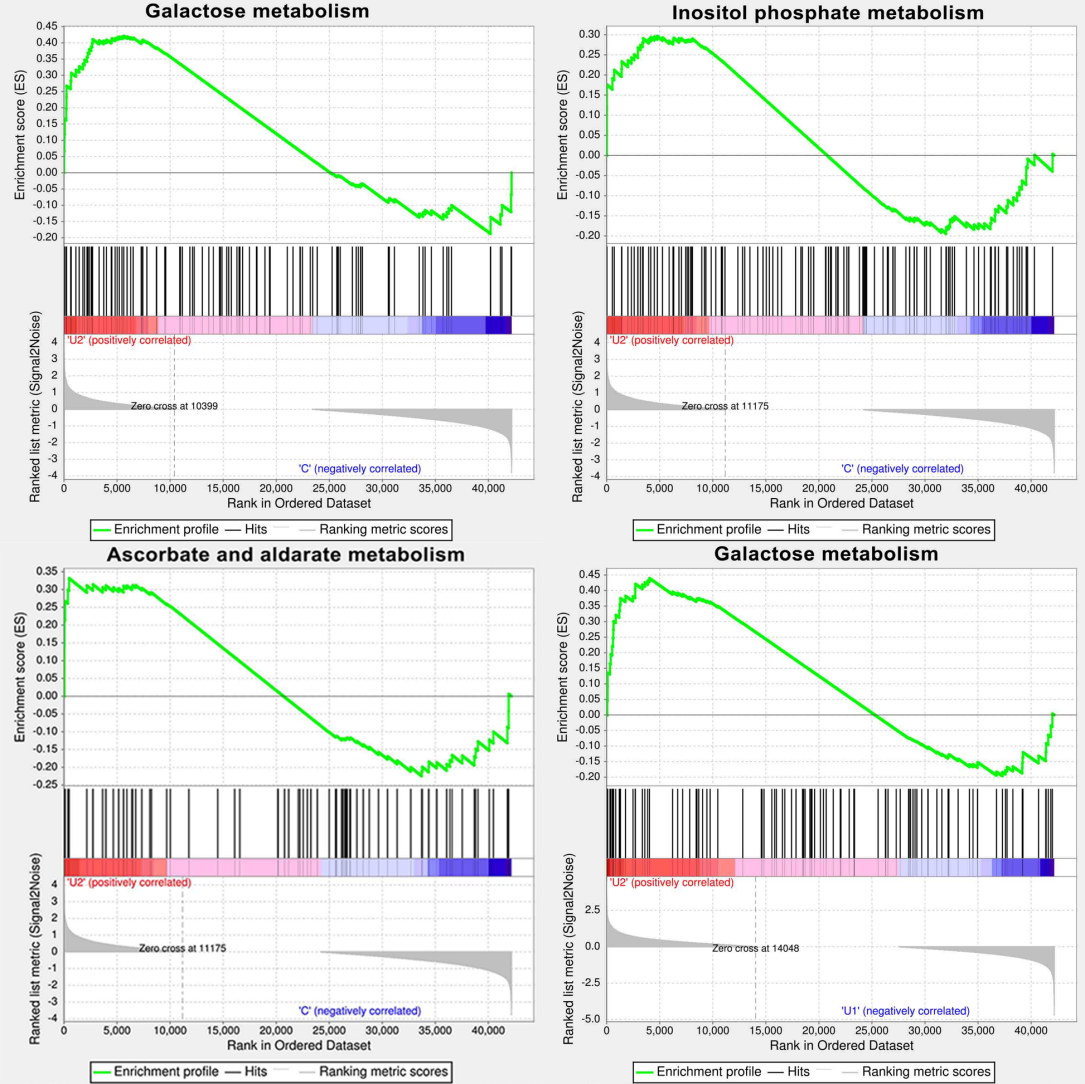
Gene set enrichment analysis (GSEA) of genes involved in ascorbic acid (AsA) biosynthesis and metabolism in lettuce

In addition, we analysed the enriched pathways (*P*-adjust < 0.5) and identified the genes that were upregulated or downregulated among the three comparisons (Additional file 3). For example, compared to C, 28 unigenes in carbohydrate metabolism, 18 unigenes in biosynthesis of secondary metabolites, 17 unigenes in signal transduction, and 10 unigenes in environmental adaptation were upregulated in U2. These enriched pathways and upregulated genes may be associated with UV-B induced AsA accumulation. We used gene set enrichment analysis (GSEA) to evaluate the differentially enriched pathways involved in AsA synthesis between treatments. The results showed that galactose metabolism, inositol phosphate metabolism, ascorbate and aldarate metabolism, starch and sucrose metabolism were enriched between C and U2, whereas only galactose metabolism was enriched between U1 and U2 (Fig. 5). These overlapping pathways, enriched in both GSEA and KEGG, warranted further analysis of the DEGs involved.

### Identification and analysis of DEGs associated with AsA under UV-B

**Fig. 6:**
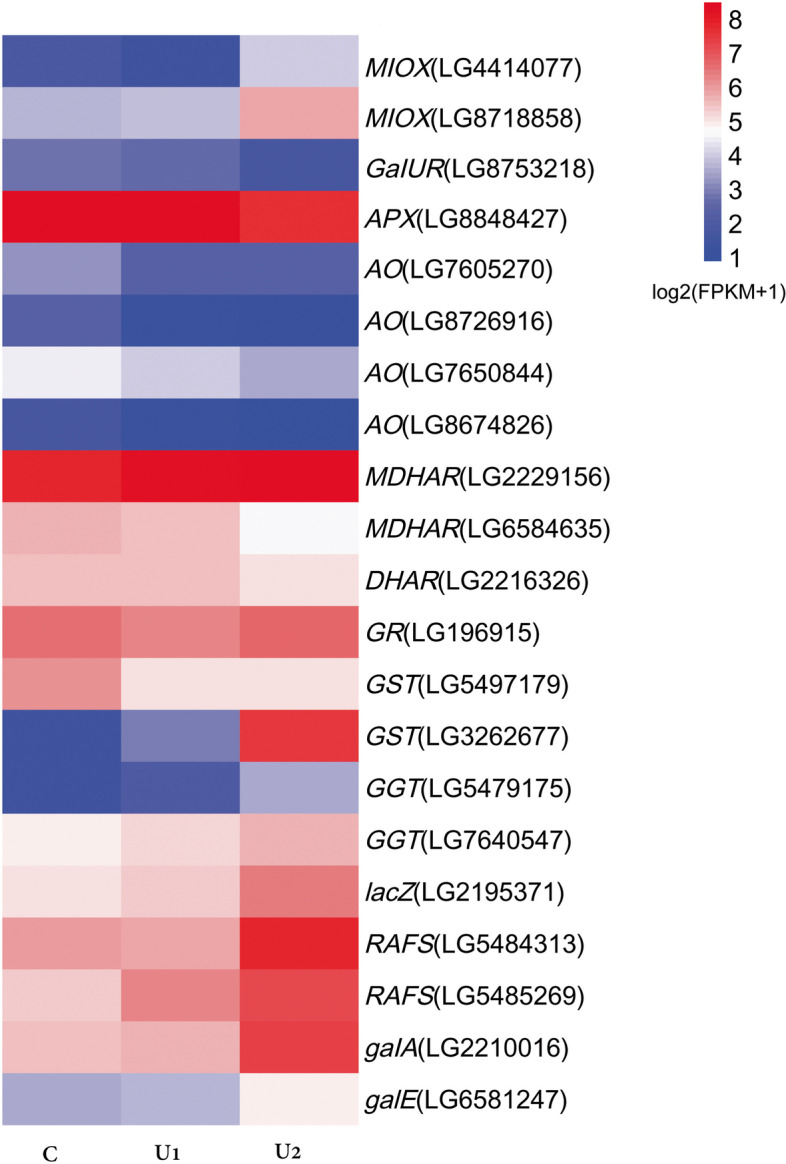
Important genes (*P*-adjust < 0.05) in ascorbic acid (AsA) biosynthesis and metabolism in lettuce

We identified 61 important unigenes in pathways associated with UV-B induced regulation of AsA levels in the three treatments, including the AsA biosynthesis pathway and recycling pathway, galactose metabolism, glutathione metabolism, photosynthesis, and respiration (Additional file 4). To understand the differences between the expression levels of these important genes among the three treatments, we screened 21 significant important genes related to AsA in U2 (*P-*adjust < 0.05; Fig. 6). *MIOX* (LG4414077, LG8718858) showed a significant increase in U2 compared to C and U1. Nine unigenes, including *APX* (one unigene), *AO* (four unigenes), *MDHAR* (two unigenes), *DHAR* (one unigene), and *GR* (one unigene), were found in the AsA recycling pathway. The expression level of *APX* (LG8848427) was significantly decreased in U2 compared to that in C and U1, whereas *AO* (LG7650844, LG8674826) was significantly decreased in U2 compared to that in C, but not significantly different compared to U1. The levels of *AO* (LG7605270, LG8726916) and *MDHAR* (LG2229156) were significantly decreased in both U1 and U2. The expression level of raffinose synthase (*RAFS*), *UDP-galactose-4-epimerase* (*galE*), *β-galactosidase* (*LacZ*), and *α-galactosidase* (*galA*)*,* which is involved in galactose metabolism, were significantly upregulated in U2, while *DHAR* (LG2216326) was significantly decreased in U2. In glutathione metabolism, the *GR*, *glutathione S-transferase* (*GST*) (LG3262677), and *γ-glutamyl transpeptidase* (*GGT1*) (LG5479175, LG7640547) expressions were significantly upregulated in U2 compared with C and U1.

### Identification and analysis of DEGs associated with hormones under UV-B

Transcriptome analysis indicated that KEGG enrichment in plant hormone signal transduction and MAPK signalling pathways differed among U2, U1, and C. We chose genes involved in hormonal pathways (Table 1). The auxin-synthesizing genes, *auxin* (*AUX*)*, small auxin upregulated* (*SAUR*)*, arabidopsis response regulator-b* (*ARR-B*) and *transport inhibitor response 1* (*TIR1*); the gibberellin synthesis pathway genes, *DELLA* and *gibberellin insensitive dwarf 2* (*GID2*)*;* and the brassinosteroid pathway genes, *brassinazole resistant1* (*BZR1*) and *cyclin D3* (*CYCD3*), were associated with growth-promoting hormones in U2, which showed the lowest significant differences (*P*-adjust < 0.05 & |log_2_FC| > = 1). However, *AUX, ARR-B, BZR1,* and *CYCD3* expression were also significantly downregulated in U1. The genes involved in both hormone synthesis and environmental stress response, including abscisic acid (ABA): sucrose nonfermenting-1 related protein kinase 2 (*SNRK2*), ABA-responsive element binding factors (*ABF*), ethylene (ETH): constitutive triple response 1 (*CTR1*), mitogen-activated protein kinase kinase 9 (*MKK9*), salicylic acid (SA): nonexpressor of pathogenesis-related genes 1 (*NPR1*), and pathogenesis resistance (*PR1*), showed the highest expression levels in U2, whereas *SNRK2, CTR1, MKK9* and *NPR1* showed no significant differences in U1.

**Table 1:** Transcript levels of hormone pathways among the three treatments

Unigene	C FPKM	U1 FPKM	U2 FPKM	Log_2_FC (CvsU1)	Log_2_FC (CvsU2)	Log_2_FC (U1vsU2)	*P*-adjust (CvsU1)	*P*-adjust (CvsU2)	*P*-adjust (U1vsU2)
*AUX* (LG1107631)	10.75	6.33	4.01	−0.66	− 1.33	− 0.67	0.02	0.003	0.31
*SAUR* (LG1132908)	7.61	9.32	2.11	0.46	−1.71	−2.17	0.59	0.02	0.00
*ARR-B* (LG3278418)	4.66	2.05	0.85	−1.07	−2.48	− 1.41	0.01	0.00	0.05
*TIR1* (LG7658414)	18.02	15.05	8.96	−0.16	−0.91	−0.75	0.69	0.00	0.06
*GH3* (LG2175806)	0.30	0.27	2.31	−0.11	3.00	3.11	0.97	0.00	0.00
*GID2* (LG6565087)	8.43	5.34	3.17	−0.57	−1.31	−0.75	0.27	0.00	0.25
*DELLA* (LG5483627)	8.52	11.61	3.09	0.53	−1.37	− 1.90	0.08	0.00	0.00
*BZR1* (LG1102677)	18.84	11.88	8.46	−0.57	−1.06	−0.50	0.04	0.00	0.34
*CYCD3* (LG3280491)	21.10	11.95	9.68	−0.73	−1.04	−0.31	0.00	0.01	0.60
*SNRK2* (LG7648102)	10.04	11.56	20.64	0.30	1.13	0.83	0.51	0.00	0.00
*SNRK2* (LG3275879)	34.42	31.10	91.10	−0.04	1.50	1.54	0.96	0.00	0.00
*SNRK2* (LG4357133)	12.49	20.64	28.37	0.82	1.28	0.46	0.00	0.00	0.01
*ABF* (LG7634878)	8.96	13.44	14.28	0.70	0.80	0.10	0.01	0.01	0.83
*CTR1* (LG1151804)	4.74	5.45	8.73	0.29	0.97	0.68	0.43	0.00	0.02
*MKK9* (LG8672927)	86.84	94.94	164.23	0.23	1.02	0.79	0.31	0.00	0.00
*NPR1* (LG5523032)	5.50	6.79	10.15	0.40	0.98	0.58	0.24	0.00	0.02
*PR1* (LG5437840)	12.90	104.16	66.37	3.13	2.49	−0.64	0.00	0.00	0.28

*Note*: *FPKM* fragments per kilobase per million reads

### qRT-PCR validation of unigenes involved in AsA metabolism from RNA-Seq

**Fig. 7:**
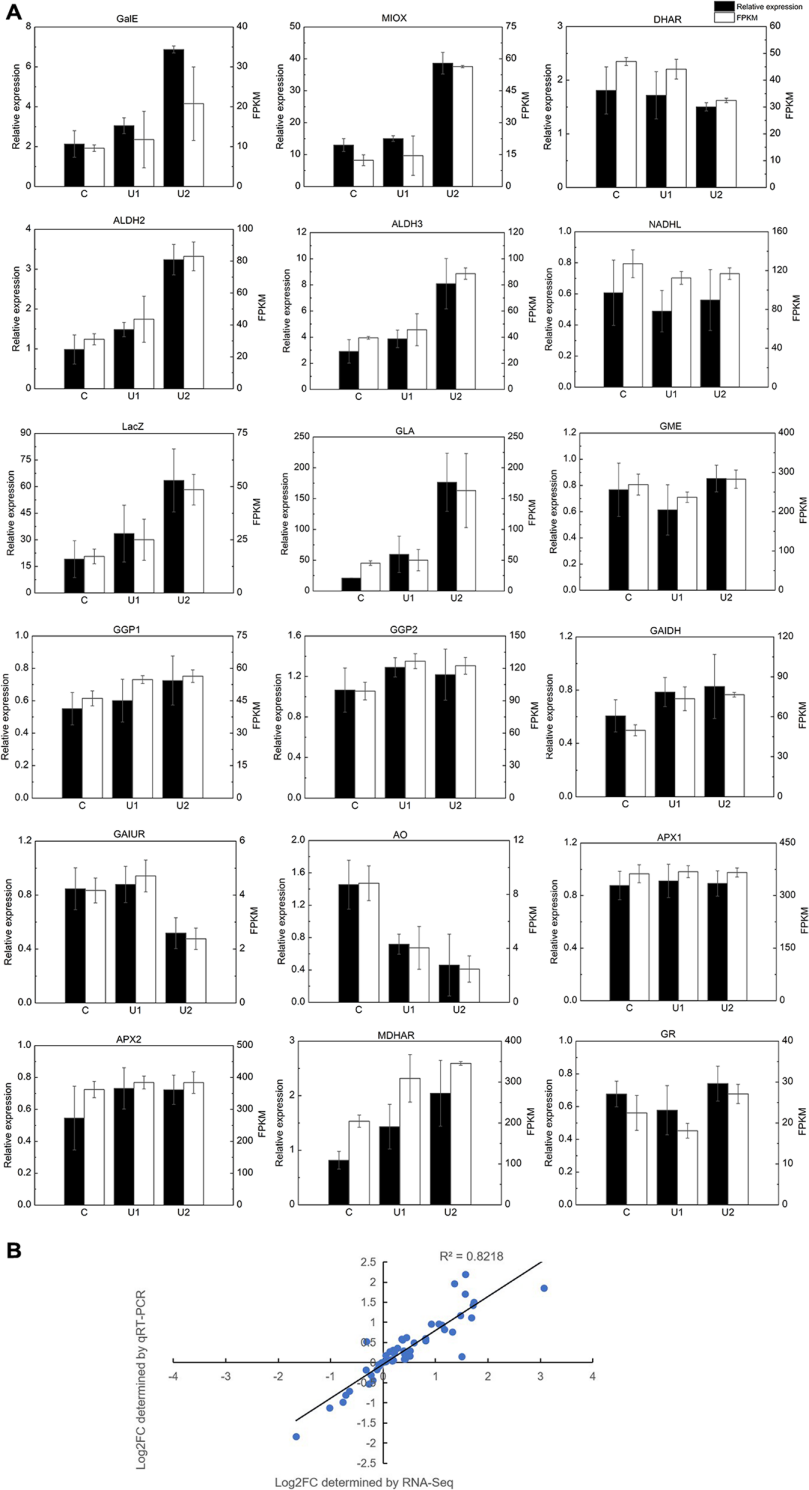
qRT-PCR validation of selected genes involved in ascorbic acid (AsA) biosynthesis of lettuce. **A**: Black bars represent the relative expression determined via qRT-PCR (left y-axis) and grey bars represent the level of expression of the transcripts (right y-axis). The error bars indicate the standard errors of three biological and three technical replicates. **B**: Scatter plots show linear regression and R^2^ between RNA-Seq and qRT-PCR in terms of log_2_FC

To validate the expression patterns of genes linked to AsA metabolism obtained from RNA-Seq, we examined the expression levels of 18 unigenes related to AsA biosynthesis (*GaIE*, *MIOX*, *DHAR*, *ALDH2*, *ALDH3*, *NADHL*, *LacZ*, *GLA*, *GME*, *GGP1*, *GGP2*, *GAIDH* and *GAIUR*) and recycling (*AO*, *APX1*, *APX2*, *MDHAR* and *GR*) in the three treatments using qRT-PCR (Fig. 7). The expression levels of these unigenes obtained via qRT-PCR were generally consistent with the data of fragments per kilobase per million reads (FPKM) using RNA-Seq. Analysis of significant differences showed that the differential expression patterns of the three treatments were similar between qRT-PCR and RNA-Seq. Moreover, the correlation between qRT-PCR and RNA-Seq was analysed and the coefficient of determination (R^2^) was 0.8218 (Fig. 7). The results confirmed the reliability of the transcriptomic profiling data from RNA-Seq measurements.

## Discussion

### High doses of UV-B inhibit growth and induce AsA accumulation in lettuce

UV-B, which acts as an environmental light signal and abiotic stress factor, affects plant development in a dose dependent manner [21, 31]. At low doses, UV-B-induced a specificity reaction wherein the light receptor, UV resistance locus 8 (UVR8) interacts with constitutively photomorphogenic 1 (COP1) active monomers, thereby inhibiting the activity of COP1 and promoting the accumulation of downstream elongated hypocotyl 5 (HY5), resulting in plant photomorphogenesis [33, 34]. At high doses, plants generate excessive oxygen stress, which damages DNA and proteins, triggering antioxidant systems to initiate defensive processes and mitigate damage [31, 34]. The effects of UV-B may be exerted independently of *UVR8*, via mechanisms regulated by hormonal pathways as reflected in photomorphogenesis and stress [35]. The hormonal regulation pathway is associated with the inhibition of growth-promoting hormones and enhancement of stress-induced defence hormones [36]. Studies have shown that leaf area, hypocotyl height, and yield of plants decreases under UV-B irradiation while leaf surface thickness increases [37, 38]. A few studies have shown that the net photosynthetic rates and yields of some plant varieties may increase under UV-B irradiation [39, 40]. In our study, the UV-B doses of 7.2 and 12.96 KJ·m^− 2^·d^− 1^, represented intermediate and high levels, respectively; there was no significant difference between the transcription levels of *UVR8* and *HY5* corresponding to the three different doses (Additional file 5). The expression pattern of genes that are associated with growth hormones was inhibited by UV-B (Table 1), which is consistent with morphogenesis induced by UV-B (Fig. 2), wherein the growth of lettuce was minimally affected at medium doses, while leaf width and shoot fresh weight were significantly inhibited at high doses. Our results indicated that the effect of UV-B on photomorphogenesis in lettuce is associated with the inhibition of growth-promoting hormones.

UV-B not only inhibits growth hormones but also increases the levels of stress response hormones [35]. ABA, considered the most important defence hormone in plants, is usually used to regulate the joint drought and UV-B stress [41]. Some studies have shown that UV-B directly induces ABA accumulation in several plants, including soybean (*Glycine max*), and maize (*Zea mays*) [42, 43]. In our study, *SNRK2* and *ABF*, associated with ABA signal transduction, were significantly upregulated under high UV-B doses and increased slightly under medium doses, indicating that UV-B may induce ABA production in lettuce in a dose-dependent manner. SA is a defence hormone that acts against biotrophs which are frequently associated with ROS production [44]. In tobacco (*Nicotiana tabacum*), UV-B increases SA by inducing *PR* which enhances plant defences [45]. Our study indicated that while *PR1* and *NPR1* were upregulated at both low and high UV-B doses, *PR1* was also significantly upregulated at low doses, showing that UV-B causes SA to accumulate rapidly, thereby reducing UV-B induced damage in lettuce. ETH, a gaseous hormone, is mainly induced by UV-B at high intensities [35]. *MKK9* was significantly upregulated only in U2 plants, indicating that high-dose UV-B increases ETH in some plant species [46].

The correlation analysis conducted by us showed that AsA was significantly negatively correlated with the number of leaves, fresh leaf weight, and dry leaf weight (data not shown). Therefore, selecting UV-B to enhance growth and quality of lettuce seems to be contradictory, because pursuing growth and yield diminishes AsA content, resulting in unknown inverse consequences for environmental adaptability [5]. In this study, there was no significant difference between growth biomasses in samples treated from 0 to 7.2 KJ·m^− 2^·d^− 1^, however, the biomass it decreased with treatments of 7.2–18.72 KJ·m^− 2^·d^− 1^. Simultaneously, lettuce AsA contents of lettuce increased gradually from 4.32–12.96 KJ·m^− 2^·d^− 1^. Thus, the dose of 7.2 KJ·m^− 2^·d^− 1^ was considered as a key dose to study the effect of UV-B on AsA content of lettuce. Therefore, a UV-B dose was 7.2–12.96 KJ·m^− 2^·d^− 1^ was recommended for use in this study to ensure both yield and AsA content.

### UV-B regulation of AsA accumulation pathways

The galactose pathway is considered the most important pathway linked to the effects exerted by light on AsA synthesis in certain plants [13, 47]. However, in this study, the expression levels of *GME, GGP, GPP, GaIDH, GLDH, and NPP* were stable, and no significant differences were observed among the three treatments, indicating that the L-galactose and glucose pathways may exert only a minimal influence on AsA accumulation in lettuce. *GaIUR* expression in lettuce decreased under UV-B irradiation, indicating that the galacturonate pathway may also exert only minimal effects on AsA synthesis in lettuce. These findings do not support the contention that light affects AsA via the galacturonate pathway [13].

Opinions differ regarding the role of *MIOX*, an important gene in the myo-inositol pathway, in AsA synthesis. Overexpression of *MIOX* in *Arabidopsis*does not increase AsA, suggesting that the myo-inositol pathway may not be the main pathway involved in AsA synthesis [48]. However, overexpression of *MIOX* rescues AsA-deficient mutants by inducing a 2–3-fold increase in AsA in *Arabidopsis*, significantly increases AsA content in transgenic tomatoes, and enhances light sensitivity of plants [11, 49]. Furthermore, *MIOX* enhances the activity of ROS-scavenging enzymes which reduce oxidative damage during drought, thereby increasing drought stress tolerance [50]. Our study showed that two *MIOX* genes were significantly upregulated in U2, indicating that *MIOX* is potentially important for AsA accumulation in lettuce under UV-B stress. Concurrently, carbohydrates act as substrates for AsA biosynthesis. Our study showed that carbohydrate metabolism is a pathway enriched in DEGs and that 14 unigenes involved in galactose and sucrose metabolism, including *RAFS, gaIE, LacZ,* and *galA*, were upregulated in U2. Moreover, changes in the expression levels of *MIOX, RAFS, gaIE, LacZ,* and *galA* were consistent with the changes in AsA content among the three treatments, leading to a significant increase in U2 with no significant difference between C and U1, indicating that these genes may be linked to AsA accumulation. Therefore, we hypothesised that *MIOX* likely responds to UV-B stress and utilises accumulated sugars to promote ASA synthesis, thereby reducing stress induced by high doses of UV-B.

The recycling pathway, which is considered a potential regulatory point for AsA synthesis [51], plays an important role in the effect exerted by light on AsA synthesis [12]. It has been suggested that the increase in the AsA contents of lettuce under a high blue light ratio is mainly attributable to the AsA recycling pathway rather than to the synthesis pathway, and the activities of enzymes involved in AsA regeneration (APX, MDHAR, DHAR, and GR) were significantly elevated and had greater correlations with the AsA level than the AsA synthesis enzyme (GLDH) [52]. Under conditions involving continuous blue light irradiation, *MDHAR* and *DHAR* play an important role in AsA accumulation in *Citrus* [53]. In this study, most genes linked to the AsA recycling pathway, including 12 *AO,* 8 *APX,* and 4 *MDHAR* genes, were found in the transcriptome of lettuce. Our results also showed that UV-B treatment induced low levels of *AO* (LG7605270, log_2_FC = − 1.08; LG8726916, log_2_FC = − 1.66; LG7650844, log_2_FC = − 0.92; LG8674826, log_2_FC = − 1.31) and *APX* (LG8848427, log_2_FC = − 0.66) while maintaining a high level of AsA in lettuce, which is consistent with the observation that inhibiting *AO* expression or the *APX* gene using RNA interference, promoted AsA accumulation in transgenic tomato plants [54, 55]. *MDHAR*, which catalyses *MDHA* to AsA, is crucial for AsA regeneration as well as for maintenance of the AsA pool. Overexpression of *MDHAR* results in AsA accumulation and promotes antioxidant capacity as well as tolerance to abiotic stress in transgenic plants [56]. In our experiment, *MDHAR* (LG2229156) expression, which remained high in U1 and U2 in response to UV-B stress (Fig. 6), maintained the AsA pool.

Likewise, the AsA-GSH cycle also plays an important role in maintaining AsA pools and removing ROS in plants [57, 58]. Compared with red light, blue light treatment effectively increased the AsA content of citrus by upregulating the GSH-producing gene *GR*, the recycling gene *DHAR*, and the biosynthetic genes *GLDH* [53]. In this study, KEGG pathway analysis indicated that, in U2, glutathione metabolism was enriched and *GR*, *GGT1*, and *GST* expression were significantly upregulated (Fig. 6), promoting the synthesis of GSH which resulted in a high level of AsA under UV-B treatment. This indicated that the AsA-GSH cycle may play an important role in AsA accumulation induced by high-dose UV-B irradiation.

**Fig. 8:**
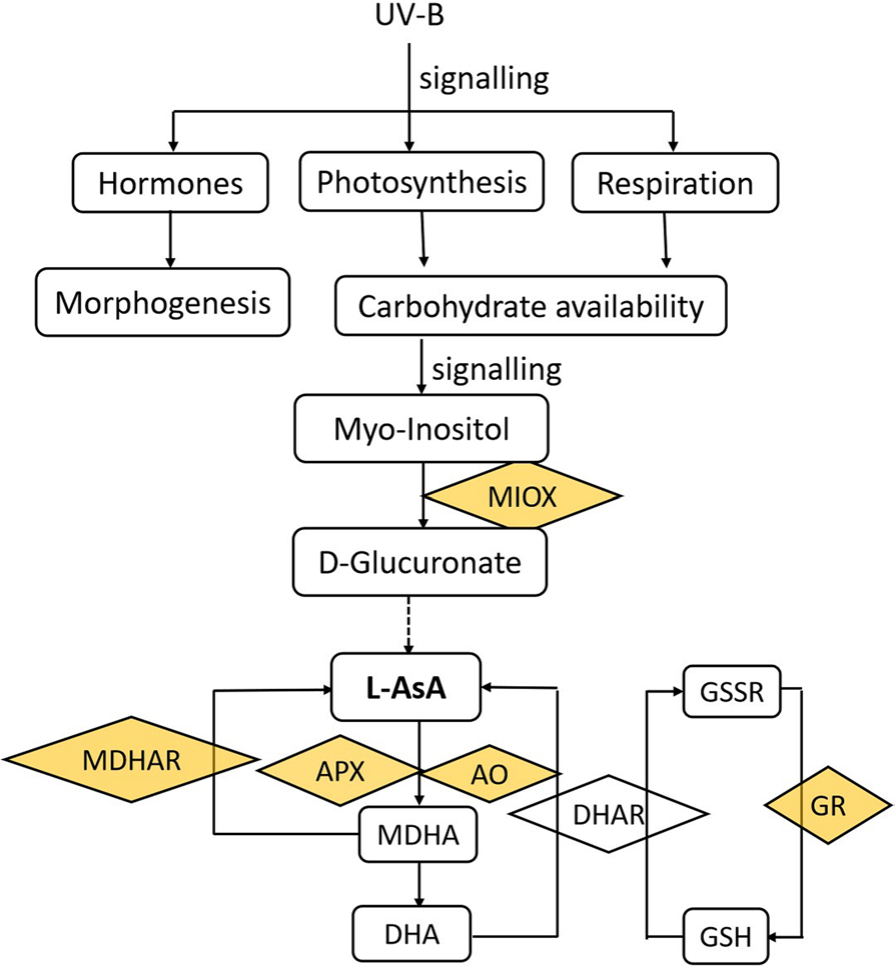
A model indicating the possible routes for AsA biosynthesis in lettuce in response to UV-B

The mechanism underlying AsA accumulation differs according to variety, organ, or developmental stage, and is regulated by multiple pathways. For example, the L-galactose pathway is responsible for ASA accumulation in tomato and grape fruits in the early stages, whereas D-galacturonic acid is responsible for same during the fruit ripening stage [59, 60]. The expression levels of genes related to AsA synthesis in the leaves, flowers, and fruits of *Citrus* plants were different. In kiwifruit (*Actinidia eriantha*), AsA synthesis is regulated by L-galactose, D-galacturonic acid, and AsA recycling pathways [61]. Our results indicated that in U2 lettuce samples, *MIOX* and *APX* were significantly highly and lowly expressed, respectively, while there was no significant difference between C and U1, which observation was consistent with ASA levels seen in these three treatments, suggesting that the myo-inositol pathway and the recycling pathway may contribute to AsA accumulation induced by UV-B in lettuce. The further function studies of the two genes are needs to elucidate the UV-B regulation mechanisms in the accumulation of AsA. A model indicating the possible routes for AsA biosynthesis in lettuce in response to UV-B treatments is presented in Fig. 8. The need to further investigate the potential of genes involved in the myo-inositol and recycling pathways is highlighted in this model.

### The role of photosynthesis and respiration in UV-B induced regulation of AsA

Light induced regulation of AsA involves the physiological processes of photosynthesis and respiration [12]. The photosynthetic rate is important for soluble carbohydrate accumulation in plants. In the AsA biosynthetic pathway, glucose acts as the initial substrate which promotes AsA levels in plants. Maintaining a highly efficient photosynthetic apparatus leads to raised AsA levels via electron donor activity involving photosystem I. Most studies have shown that UV-B irradiation causes a reduction in photosynthetic activity, damages Photosystem II proteins, and destroys chlorophyll and carotenoids, resulting in reduced crop yield [62, 63]. However, other studies have suggested that acclimation to UV-B radiation increases the net photosynthetic rate and upregulates secondary metabolite production [39, 64]. The results of the present study indicated that some genes involved in the photosystem, such as *apocytochrome f* (*petA*)*, cytochrome b6* (*petB*)*, and photosystem I P700 chlorophyll a apoprotein A2* (*PsaB*) (Additional file 4), were highly expressed in U1 and U2, whereas carbohydrate metabolism and secondary metabolites were enriched under UV-B treatment (Additional file 3). This suggests that UV radiation does not limit photosynthetic efficiency, and that secondary metabolites may effectively protect the photosynthetic apparatus [65]. Increasing photosynthetic competency can promote carbohydrate availability, resulting in high AsA levels, although growth was significantly inhibited by the U2 treatment.

AsA biosynthesis was proven to be regulated by the mitochondrial respiratory chain [66]. GLDH acts as a link between AsA biosynthesis and respiration [67]. A study revealed that interaction between light and respiration modulates GLDH activity and thereby regulates AsA synthesis [68]. Alternative oxidase (AOX), a terminal oxidase of the mitochondrial electron transport chain, is involved in cell respiration as well as in photosynthesis and plays an important role in light stress tolerance. Overexpression of *AOX* in *Arabidopsis* promotes AsA accumulation [69]. In this study, *GLDH* showed no significant differences among the three treatments, but three *AOX1* genes were significantly upregulated in U2 (Additional file 4), suggesting that AOX respiratory pathways may affect AsA biosynthesis.

## Conclusion

We investigated the effect of UV-B on AsA in lettuce, and the results revealed a relationship between UV-B dose and AsA accumulation. No UV-B and low dose of UV-B were clustered into one category, whereas the high dose of UV-B was clustered into another. Transcriptome data revealed the processes associated with AsA metabolism in lettuce, where 61 genes related to AsA synthesis and metabolism were identified at medium and high doses of UV-B. Changes in the expression levels of *MIOX* and *APX* among the three treatments were consistent with fluctuations in AsA levels, and the genes involved in carbohydrate, inositol phosphate and glutathione metabolism pathways were significantly upregulated in U2, suggesting that the myo-inositol and recycling pathways may contribute to AsA accumulation. The effects of UV-B in lettuce may be independent of the UVR8 pathway; and the hormonal signalling pathway, including growth-inhibition hormones and stress hormones, may regulate photomorphology and stress response. Both yield and AsA content should be taken into consideration when deciding on the dose of UV-B to be applied, and the recommended dose in this study is 7.2–12.96 KJ·m^− 2^·d^− 1^. In addition, photosynthesis and respiration were found to affect UV-B regulation of AsA. The findings of this study may lead to the development of a useful approach for regulating the growth, development, and AsA contents of lettuce via UV-B irradiation. The discovery of genes associated with AsA and hormones involved in UV-B signalling in lettuce is worthy of further investigation. Quantitative transcriptomics and metabolomics study of UV-B induced AsA accumulation are needed to provide a more comprehensive view and genetic framework of these regulation pathways for AsA biosynthesis and metabolism in lettuce.

## Methods

### Plant materials and treatments

Green leaf lettuce (*Lactuca sativa* L. cv. ‘green rose’) seeds were provided by Shanghai Wells Seed Co., Ltd. in Shanghai (China) and were grown in a plant factory at the Jiangxi Academy of Sciences in Nanchang, Jiangxi Province, China. Seedlings that had reached the 2–3 leaf stage were transplanted in a plant factory. Conditions in the plant factory were set to 20–22 °C, 60–80% humidity, and a 400 mg·L^− 1^ CO_2_ concentration. The potted lettuce plants were supplemented with the Hoagland nutrient solution [70] every 5 days. The light source for each layer was provided using LED tubes (Jinghe Lighting Co., Ltd., Nanchang, China). Light quality consisted of red light (R) and blue light (B) at a ratio of 2:1, where the wavelengths of red light and blue light were 660 ± 10 nm, and 450 ± 10 nm, respectively. The illumination time and photosynthetic photon flux density was 18 h per day and 200 μmol·m^− 2^ s^− 1^, respectively.

Irradiation was performed using UV-B lamps (Phillips TL 100 W/01, Eindhoven, The Netherlands) Doses of 0 (control), 20, 60, 100, 140, 180, 220, and 260 mW·m^− 2^ plant weighted UV-B was applied for a 2-h exposure leading to a total irradiation of 0, 1.44, 4.32, 7.20, 10.08, 12.96, 15.84, and 18.72 kJ·m^−2^d^− 1^. UV-B dose was determined by adjusting the distance between the tube and the plant, and measured using an LS 125 UV light meter host (Linshang Technology Co., Ltd., Shenzhen, China). Lettuce was investigated for growth and AsA content 20 d later, and leaf samples were collected for transcriptome sequencing.

### Growth measurements and AsA content

Plant growth parameters, such as the number of leaves, stem diameter, leaf length, leaf width, and fresh and dry weights of the plants were measured. Eight randomly selected plants were used to evaluate growth parameters for each treatment on Day 20 after transplantation. All leaves with leaf lengths greater than 2 cm were included in leaf number counts, whereas the diameter of the plant stem up to 1 cm from the root was considered as the stem diameter. The average length and width of the three largest blades were considered as leaf length and width, respectively. The shoots and roots of lettuce were cut, removed, and used to determine shoot fresh weight and root fresh weight, respectively. Next, all shoots and roots were oven dried at 105 °C for 5 min and again dried at 70 °C to obtain constant dry weight.

Fresh leaves without petioles obtained from three randomly selected plants were collected from each treatment on transplantation Day 19. The collected leaves were immediately frozen in liquid nitrogen and stored in an ultra-low temperature freezer (− 80 °C) for AsA measurement and transcriptome analysis. The AsA concentrations of the three biological replicates were determined using a spectrophotometer (METASH UV-5100, Shanghai, China), according to the method described by Gillespie and Ainsworth [71].

### RNA isolation, library preparation, and transcriptome sequencing

The samples subjected to UV-B doses of 0, 7.2, and 12.96 KJ·m^− 2^·d^− 1^ were used for total RNA extraction and RNA-Seq analysis. Total RNA was extracted using the Trizol reagent kit (Ivtitrogen, Carlsbad, CA, USA). RNA quality was assessed by the Agilent Bioanalyzer 2100 system (Agilent Technologies, CA, USA) and agarose gel electrophoresis. Eukaryotic mRNA was isolated from total RNA, fragmented into short fragments, and reverse transcribed into cDNA for transcriptome analysis using Illumina TruseqTM RNA sample prep Kit (Illumina San Diego, CA, USA). The cDNA library was sequenced using the NovaSeq 6000 sequencing platform (Illumina).

### Mapping, annotation, and analysis of unigenes

Based on the reference genome information for lettuce, the TopHat2 software (http://ccb.jhu.edu/software/tophat/index.shtml) [72] was used for sequence alignment analysis and the StringTiesoftware (http://ccb.jhu.edu/software/stringtie/) [32] was used for assembly splicing of mapped reads. The mapped reads were compared with the original genome annotation information to find unannotated transcripts as well as to discover new transcripts and genes of lettuce species. Genes/transcripts were compared with six databases: Nr (http://ftp.ncbi.nlm.nih.gov/blast/db/), Swiss-Prot (http://web.expasy.org/docs/swiss-prot_guideline.html), Pfam (http://pfam.xfam.org/), EggNOG (http://www.ncbi.nlm.nih.gov/COG/), GO (http://www.geneontology.org), and KEGG (http://www.genome.jp/kegg/) to obtain the annotation information of genes/transcripts. The number of FPKM was used to estimate the relative expression levels. The expression levels of genes and transcripts were quantitatively analysed using the RSEM software (http://deweylab.github.io/RSEM/) [73]. Differential expression analysis of samples was performed using the DESeq2 and the *P*-values were adjusted to *P*-adjust to compensate of multiple hypothesis testing. Genes with *P*-adjust < 0.05 & |log_2_FC| > = 1 were assigned as differentially expressed and the fold change (FC) was calculated as the ratio between samples. GO enrichment analysis of differentially expressed genes were performed with Goatools (https://github.com/tanghaibao/GOatools) [74]. KEGG pathway enrichment analysis was performed using the R package [75]. GSEA, to comp5are differentially enriched pathways between samples, was conducted using GSEA software (http://software.broadinstitute.org/gsea/index/jsp) [76]. The item annotated gene set was chosen as the reference gene list, and results with a cut-off criterion of *P*-adjust < 0.05 were considered statistically significant.

### qRT-PCR verification

After extracting genomic DNA, cDNA was synthesised using an iScript™ gDNA Clear cDNA Synthesis Kit (Bio-Rad, Hercules, CA, USA). Using the SYBR Green PCR Master Mix (Takara, Dalian, China) and ABI ViiA real-time PCR platform, 18 novel transcripts related to ascorbic acid biosynthesis were selected for gene expression analysis. Specific primers were designed using Oligo 7.0 (Molecular Biology Insights, Cascade, CO, USA), the details of which are listed (Additional file 6). Three technical replicates were analysed via PCR. The PCR mixture contained 0.5 μL of cDNA, 10 μL of SYBR Green PCR Master Mix, 8.5 μL of ddH_2_O, and 10 μM primers with a final 20 μL per reaction. Glyceraldehyde-3-phosphate dehydrogenase (*GAPDH*) in lettuce was considered as the control gene for normalisation, and relative expression levels were calculated using the 2^-∆∆ Ct^ method.

### Statistical analysis

SPSS 20.0 (IBM, Armonk, NY, USA) was used for growth data analysis. The mean and standard deviation were calculated. The data was analysed using one-way analysis of variance, and Duncan’s new complex range method was used for multiple comparisons.

Raw counts were analysed for differential expression using the DESeq2 (http://www.bioconductor.org/packages/release/bioc/html/DESeq2.html) [77] software based on a negative binomial distribution, and genes/transcripts with DEGs between groups were obtained based on standardised treatment and screening conditions.

## Supplementary Information

**Additional file 1: Figure S1.** Correlation analysis between samples. The right and lower sides are samples names, the left and upper sides are samples cluster, and squares of different colors represent correlation between the two samples.

**Additional file 2: Figure S2.** Principal component analysis (PCA) of samples.

**Additional file 3: Table S1.** The enrichment pathway among the three comparisons.

**Additional file 4: Table S2.** Unigenes involved in AsA biosynthesis and metabolism in lettuce.

**Additional file 5: Table S3.** Transcript levels of UVR8 and HY5 in the three treatments.

**Additional file 6: Table S4.** Primers for real time RT-PCR analysis.

## Data Availability

The datasets generated or analyzed during the current study (raw RNA-Seq reads) will be available in the National Center for Biotechnology Information (NCBI) Sequence Read Archive (SRA) after the release data (2023-09-01): PRJNA869133，https://www.ncbi.nlm.nih.gov/sra/PRJNA869133.

## Abbreviations

AsA: Vitamin C (L-ascorbic acid)
Nr: Non-redundant proteins sequence database
Swissprot: Annotated protein sequence database
Pfam: The protein families database
EggNOG: Clusters of orthologous groups of proteins
GO: Gene ontology
KEGG: Kyoto encyclopedia of genes and genomes
FPKM: Fragments per kilobase per million reads
DEGs: Differentially expressed genes
qRT-PCR: quantitative reverse transcription PCR
RNA-Seq: Transcriptome sequencing
SA: Salicylic acid
ETH: Ethylene
PR: Pathogenesis resistance
