# Supplementary figures and images for: Transcriptome comparison analyses in UV-B induced AsA accumulation of Lactuca sativa L
Source: BMC Genomics. 2023 Feb 3;24:61. doi: 10.1186/s12864-023-09133-7 (PMC9896689; doi:10.1186/s12864-023-09133-7)

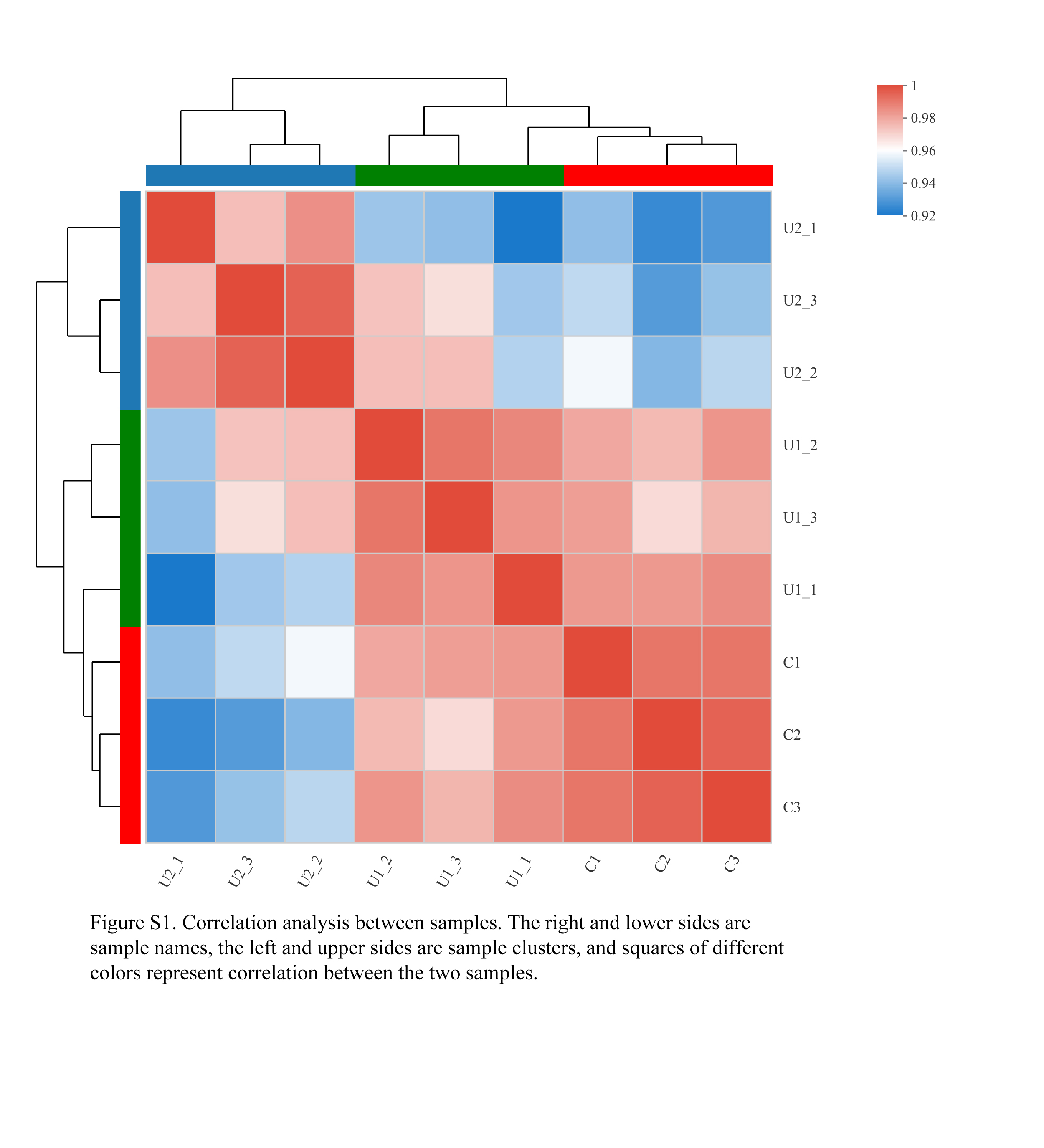

Supplement: Supplementary file 1 — Additional file 1: Figure S1. Correlation analysis between samples. The right and lower sides are samples names, the left and upper sides are samples cluster, and squares of different colors represent correlation between the two samples. [file 12864_2023_9133_MOESM1_ESM.jpg]

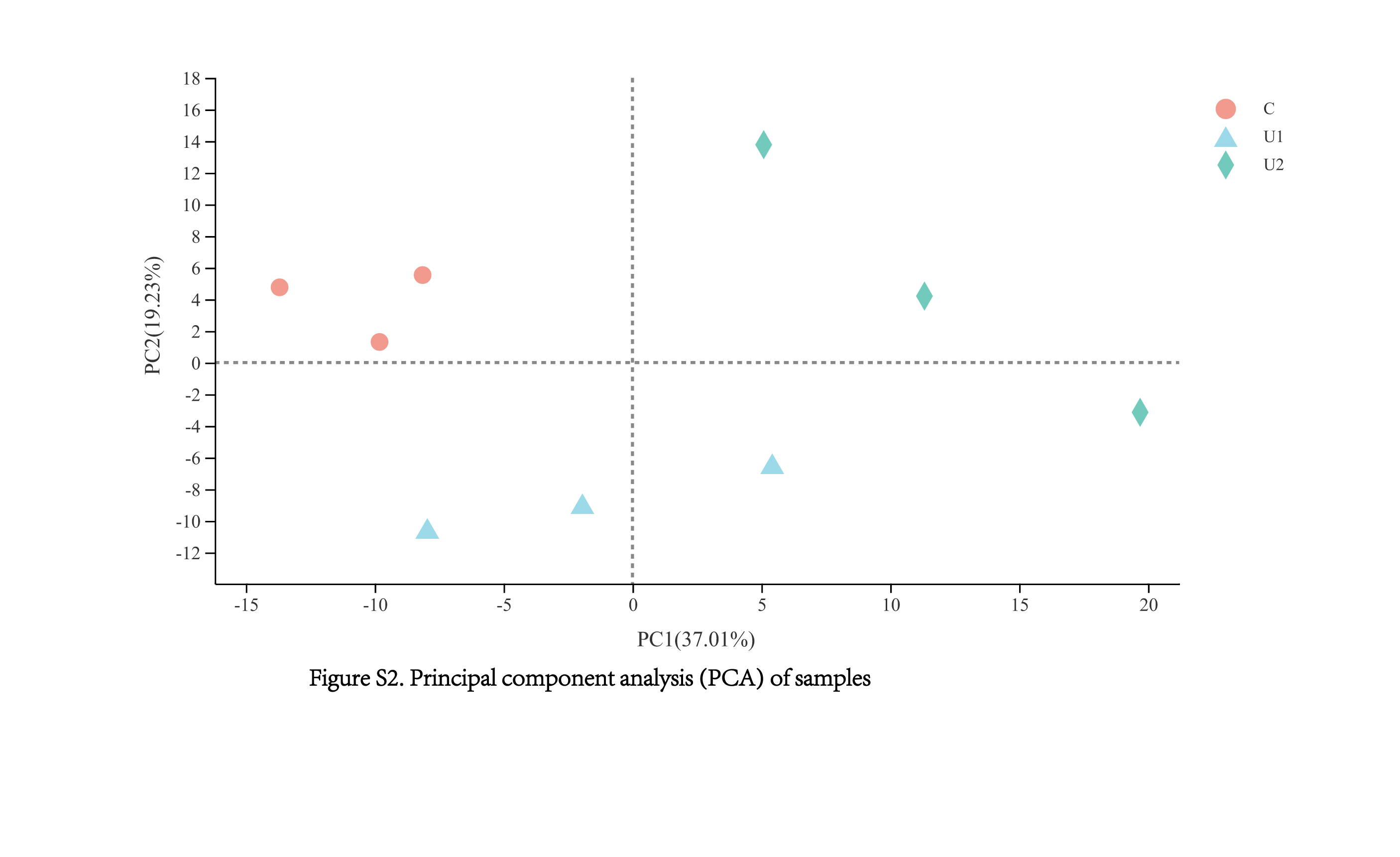

Supplement: Supplementary file 2 — Additional file 2: Figure S2. Principal component analysis (PCA) of samples. [file 12864_2023_9133_MOESM2_ESM.jpg]
