# Supplementary material for: Transcriptome comparison analyses in UV-B induced AsA accumulation of Lactuca sativa L
Source: BMC Genomics. 2023 Feb 3;24:61. doi: 10.1186/s12864-023-09133-7 (PMC9896689; doi:10.1186/s12864-023-09133-7)
Supplement: Supplementary file 3 — Additional file 3: Table S1. The enrichment pathway among the three comparisons. [file 12864_2023_9133_MOESM3_ESM.doc]

**Table S1 T**he enrichment pathway among the three comparisons

| **Comprison** | **Regula-tion** | **Num-ber** | **Description** | **P-value** | **P-adjust** | **First Category** | **Second Category** |
| --- | --- | --- | --- | --- | --- | --- | --- |
| C vs U2 | up | 6 | Phenylalanine metabolism | 0.0001 | 0.0023 | Metabolism | Amino acid meta |
| C vs U2 | up | 8 | Valine, leucine and isoleucine degradation | 0.0000 | 0.0025 | Metabolism | Amino acid meta |
| C vs U2 | up | 7 | Galactose metabolism | 0.0003 | 0.0077 | Metabolism | Carbohydrate metabolism |
| C vs U2 | up | 5 | Tropane, piperidine and pyridine alkaloid biosynthesis | 0.0006 | 0.0129 | Metabolism | Biosynthesis of other secondary metabolites |
| C vs U2 | up | 6 | Tyrosine metabolism | 0.0013 | 0.0164 | Metabolism | Amino acid metabolism |
| C vs U2 | up | 5 | beta-Alanine metabolism | 0.0010 | 0.0165 | Metabolism | Metabolism of other amino acids |
| C vs U2 | up | 5 | Biosynthesis of unsaturated fatty acids | 0.0012 | 0.0175 | Metabolism | Lipid metabolism |
| C vs U2 | up | 4 | Isoquinoline alkaloid biosynthesis | 0.0026 | 0.0276 | Metabolism | Biosynthesis of other secondary metabolites |
| C vs U2 | up | 3 | Monoterpenoid biosynthesis | 0.0047 | 0.0448 | Metabolism | Metabolism of terpenoids and polyketides |
| C vs U2 | up | 2 | Limonene and pinene degradation | 0.0058 | 0.0495 | Metabolism | Metabolism of terpenoids and polyketides |
| C vs U2 | up | 6 | Glycerolipid metabolism | 0.0069 | 0.0533 | Metabolism | Lipid metabolism |
| C vs U2 | up | 2 | Brassinosteroid biosynthesis | 0.0195 | 0.1275 | Metabolism | Metabolism of terpenoids and polyketides |
| C vs U2 | up | 4 | Alanine, aspartate and glutamate metabolism | 0.0194 | 0.1372 | Metabolism | Amino acid metabolism |
| C vs U2 | up | 7 | MAPK signaling pathway - plant | 0.0268 | 0.1628 | Environmental Information Processing | Signal transduction |
| C vs U2 | up | 7 | Starch and sucrose metabolism | 0.0343 | 0.1822 | Metabolism | Carbohydrate metabolism |
| C vs U2 | up | 3 | Sphingolipid metabolism | 0.0379 | 0.1895 | Metabolism | Lipid metabolism |
| C vs U2 | up | 10 | Plant hormone signal transduction | 0.0342 | 0.1939 | Environmental Information Processing | Signal transduction |
| C vs U2 | up | 2 | Butanoate metabolism | 0.0515 | 0.2086 | Metabolism | Carbohydrate metabolism |
| C vs U2 | up | 3 | Phenylalanine, tyrosine and tryptophan biosynthesis | 0.0446 | 0.2106 | Metabolism | Amino acid metabolism |
| C vs U2 | up | 2 | Arachidonic acid metabolism | 0.0474 | 0.2121 | Metabolism | Lipid metabolism |
| C vs U2 | up | 4 | Fatty acid degradation | 0.0506 | 0.2151 | Metabolism | Lipid metabolism |
| C vs U2 | up | 3 | Flavonoid biosynthesis | 0.0569 | 0.2199 | Metabolism | Biosynthesis of other secondary metabolites |
| C vs U2 | up | 3 | Tryptophan metabolism | 0.0596 | 0.2201 | Metabolism | Amino acid metabolism |
| C vs U2 | up | 3 | Ascorbate and aldarate metabolism | 0.0650 | 0.2303 | Metabolism | Carbohydrate metabolism |
| C vs U2 | up | 3 | Lysine degradation | 0.0707 | 0.2404 | Metabolism | Amino acid metabolism |
| C vs U2 | up | 4 | Carbon fixation in photosynthetic organisms | 0.0761 | 0.2487 | Metabolism | Energy metabolism |
| C vs U2 | up | 6 | Phenylpropanoid biosynthesis | 0.0816 | 0.2570 | Metabolism | Biosynthesis of other secondary metabolites |
| C vs U2 | up | 4 | Glycine, serine and threonine metabolism | 0.0878 | 0.2664 | Metabolism | Amino acid metabolism |
| C vs U2 | up | 7 | Plant-pathogen interaction | 0.0980 | 0.2873 | Organismal Systems | Environmental adaptation |
| C vs U2 | up | 3 | Circadian rhythm - plant | 0.1022 | 0.2895 | Organismal Systems | Environmental adaptation |
| C vs U2 | up | 3 | Propanoate metabolism | 0.1108 | 0.3038 | Metabolism | Carbohydrate metabolism |
| C vs U2 | up | 2 | Sesquiterpenoid and triterpenoid biosynthesis | 0.1147 | 0.3046 | Metabolism | Metabolism of terpenoids and polyketides |
| C vs U2 | up | 3 | alpha-Linolenic acid metabolism | 0.1215 | 0.3129 | Metabolism | Lipid metabolism |
| C vs U2 | up | 4 | Cysteine and methionine metabolism | 0.1290 | 0.3133 | Metabolism | Amino acid metabolism |
| C vs U2 | up | 3 | Fructose and mannose metabolism | 0.1288 | 0.3220 | Metabolism | Carbohydrate metabolism |
| C vs U2 | up | 1 | Glycosphingolipid biosynthesis - globo and isoglobo series | 0.1604 | 0.3788 | Metabolism | Glycan biosynthesis and metabolism |
| C vs U2 | up | 2 | Arginine biosynthesis | 0.1743 | 0.3800 | Metabolism | Amino acid metabolism |
| C vs U2 | up | 1 | Non-homologous end-joining | 0.1743 | 0.4004 | Genetic Information Processing | Replication and repair |
| C vs U2 | up | 1 | Taurine and hypotaurine metabolism | 0.1743 | 0.4004 | Metabolism | Metabolism of other amino acids |
| C vs U2 | up | 3 | Inositol phosphate metabolism | 0.2093 | 0.4447 | Metabolism | Carbohydrate metabolism |
| C vs U2 | up | 2 | ABC transporters | 0.2469 | 0.4997 | Environmental Information Processing | Membrane transport |
| C vs U2 | down | 8 | Glycerolipid metabolism | 0.0003 | 0.0127 | Metabolism | Lipid metabolism |
| C vs U2 | down | 10 | Starch and sucrose metabolism | 0.0012 | 0.0305 | Metabolism | Carbohydrate metabolism |
| C vs U2 | down | 4 | Isoquinoline alkaloid biosynthesis | 0.0027 | 0.0535 | Metabolism | Biosynthesis of other secondary metabolites |
| C vs U2 | down | 4 | Fatty acid elongation | 0.0037 | 0.0579 | Metabolism | Lipid metabolism |
| C vs U2 | down | 5 | Tyrosine metabolism | 0.0077 | 0.1006 | Metabolism | Amino acid metabolism |
| C vs U2 | down | 2 | Monobactam biosynthesis | 0.0172 | 0.1918 | Metabolism | Biosynthesis of other secondary metabolites |
| C vs U2 | down | 2 | Lysine biosynthesis | 0.0200 | 0.1954 | Metabolism | Amino acid metabolism |
| C vs U2 | down | 4 | Pyrimidine metabolism | 0.0262 | 0.2040 | Metabolism | Nucleotide metabolism |
| C vs U2 | down | 1 | Biosynthesis of various secondary metabolites - part 2 | 0.0250 | 0.2169 | Metabolism | Biosynthesis of other secondary metabolites |
| C vs U2 | down | 1 | Mannose type O-glycan biosynthesis | 0.0494 | 0.2966 | Metabolism | Glycan biosynthesis and metabolism |
| C vs U2 | down | 5 | Cysteine and methionine metabolism | 0.0475 | 0.3089 | Metabolism | Amino acid metabolism |
| C vs U2 | down | 1 | Flavone and flavonol biosynthesis | 0.0574 | 0.3199 | Metabolism | Biosynthesis of other secondary metabolites |
| C vs U2 | down | 8 | Plant-pathogen interaction | 0.0465 | 0.3295 | Organismal Systems | Environmental adaptation |
| C vs U2 | down | 6 | Phenylpropanoid biosynthesis | 0.0862 | 0.3953 | Metabolism | Biosynthesis of other secondary metabolites |
| C vs U2 | down | 5 | Glycerophospholipid metabolism | 0.0775 | 0.4032 | Metabolism | Lipid metabolism |
| C vs U2 | down | 3 | Cyanoamino acid metabolism | 0.0855 | 0.4170 | Metabolism | Metabolism of other amino acids |
| C vs U2 | down | 2 | Zeatin biosynthesis | 0.1175 | 0.4822 | Metabolism | Metabolism of terpenoids and polyketides |
| C vs U2 | down | 1 | Limonene and pinene degradation | 0.1116 | 0.4835 | Metabolism | Metabolism of terpenoids and polyketides |
| C vs U1 | up | 2 | Nitrogen metabolism | 0.0119 | 0.0955 | Metabolism | Energy metabolism |
| C vs U1 | up | 3 | Glutathione metabolism | 0.0101 | 0.1209 | Metabolism | Metabolism of other amino acids |
| C vs U1 | up | 2 | Flavonoid biosynthesis | 0.0204 | 0.1226 | Metabolism | Biosynthesis of other secondary metabolites |
| C vs U1 | up | 2 | Sesquiterpenoid and triterpenoid biosynthesis | 0.0095 | 0.2273 | Metabolism | Metabolism of terpenoids and polyketides |
| C vs U1 | up | 2 | Peroxisome | 0.0906 | 0.4349 | Cellular Processes | Transport and catabolism |
| C vs U1 | up | 1 | Carotenoid biosynthesis | 0.1089 | 0.4358 | Metabolism | Metabolism of terpenoids and polyketides |
| C vs U1 | up | 1 | Porphyrin and chlorophyll metabolism | 0.2228 | 0.4455 | Metabolism | Metabolism of cofactors and vitamins |
| C vs U1 | up | 1 | Circadian rhythm - plant | 0.2469 | 0.4559 | Organismal Systems | Environmental adaptation |
| C vs U1 | up | 2 | Phenylpropanoid biosynthesis | 0.1753 | 0.4674 | Metabolism | Biosynthesis of other secondary metabolites |
| C vs U1 | up | 1 | ABC transporters | 0.2145 | 0.4681 | Environmental Information Processing | Membrane transport |
| C vs U1 | up | 1 | Zeatin biosynthesis | 0.1366 | 0.4683 | Metabolism | Metabolism of terpenoids and polyketides |
| C vs U1 | up | 2 | MAPK signaling pathway - plant | 0.1656 | 0.4969 | Environmental Information Processing | Signal transduction |
| C vs U1 | down | 2 | Nitrogen metabolism | 0.0119 | 0.0955 | Metabolism | Energy metabolism |
| C vs U1 | down | 3 | Glutathione metabolism | 0.0101 | 0.1209 | Metabolism | Metabolism of other amino acids |
| C vs U1 | down | 2 | Flavonoid biosynthesis | 0.0204 | 0.1226 | Metabolism | Biosynthesis of other secondary metabolites |
| C vs U1 | down | 2 | Sesquiterpenoid and triterpenoid biosynthesis | 0.0095 | 0.2273 | Metabolism | Metabolism of terpenoids and polyketides |
| C vs U1 | down | 2 | Peroxisome | 0.0906 | 0.4349 | Cellular Processes | Transport and catabolism |
| C vs U1 | down | 1 | Carotenoid biosynthesis | 0.1089 | 0.4358 | Metabolism | Metabolism of terpenoids and polyketides |
| C vs U1 | down | 1 | Porphyrin and chlorophyll metabolism | 0.2228 | 0.4455 | Metabolism | Metabolism of cofactors and vitamins |
| C vs U1 | down | 1 | Circadian rhythm - plant | 0.2469 | 0.4559 | Organismal Systems | Environmental adaptation |
| C vs U1 | down | 2 | Phenylpropanoid biosynthesis | 0.1753 | 0.4674 | Metabolism | Biosynthesis of other secondary metabolites |
| C vs U1 | down | 1 | ABC transporters | 0.2145 | 0.4681 | Environmental Information Processing | Membrane transport |
| C vs U1 | down | 1 | Zeatin biosynthesis | 0.1366 | 0.4683 | Metabolism | Metabolism of terpenoids and polyketides |
| C vs U1 | down | 2 | MAPK signaling pathway - plant | 0.1656 | 0.4969 | Environmental Information Processing | Signal transduction |
| U1 vs U2 | up | 6 | Valine, leucine and isoleucine degradation | 0.0000 | 0.0013 | Metabolism | Amino acid metabolism |
| U1 vs U2 | up | 5 | Biosynthesis of unsaturated fatty acids | 0.0001 | 0.0013 | Metabolism | Lipid metabolism |
| U1 vs U2 | up | 5 | Phenylalanine metabolism | 0.0000 | 0.0016 | Metabolism | Amino acid metabolism |
| U1 vs U2 | up | 5 | Tryptophan metabolism | 0.0001 | 0.0016 | Metabolism | Amino acid metabolism |
| U1 vs U2 | up | 4 | Isoquinoline alkaloid biosynthesis | 0.0002 | 0.0028 | Metabolism | Biosynthesis of other secondary metabolites |
| U1 vs U2 | up | 5 | Tyrosine metabolism | 0.0004 | 0.0044 | Metabolism | Amino acid metabolism |
| U1 vs U2 | up | 5 | Galactose metabolism | 0.0005 | 0.0045 | Metabolism | Carbohydrate metabolism |
| U1 vs U2 | up | 2 | Brassinosteroid biosynthesis | 0.0057 | 0.0379 | Metabolism | Metabolism of terpenoids and polyketides |
| U1 vs U2 | up | 3 | Tropane, piperidine and pyridine alkaloid biosynthesis | 0.0055 | 0.0414 | Metabolism | Biosynthesis of other secondary metabolites |
| U1 vs U2 | up | 3 | Phenylalanine, tyrosine and tryptophan biosynthesis | 0.0083 | 0.0497 | Metabolism | Amino acid metabolism |
| U1 vs U2 | up | 3 | Ascorbate and aldarate metabolism | 0.0126 | 0.0689 | Metabolism | Carbohydrate metabolism |
| U1 vs U2 | up | 3 | Lysine degradation | 0.0139 | 0.0694 | Metabolism | Amino acid metabolism |
| U1 vs U2 | up | 3 | Circadian rhythm - plant | 0.0212 | 0.0848 | Organismal Systems | Environmental adaptation |
| U1 vs U2 | up | 4 | Cysteine and methionine metabolism | 0.0190 | 0.0875 | Metabolism | Amino acid metabolism |
| U1 vs U2 | up | 5 | Phenylpropanoid biosynthesis | 0.0211 | 0.0903 | Metabolism | Biosynthesis of other secondary metabolites |
| U1 vs U2 | up | 3 | Fatty acid degradation | 0.0365 | 0.1289 | Metabolism | Lipid metabolism |
| U1 vs U2 | up | 2 | Selenocompound metabolism | 0.0364 | 0.1365 | Metabolism | Metabolism of other amino acids |
| U1 vs U2 | up | 2 | beta-Alanine metabolism | 0.0598 | 0.1630 | Metabolism | Metabolism of other amino acids |
| U1 vs U2 | up | 3 | Inositol phosphate metabolism | 0.0502 | 0.1674 | Metabolism | Carbohydrate metabolism |
| U1 vs U2 | up | 1 | Limonene and pinene degradation | 0.0592 | 0.1692 | Metabolism | Metabolism of terpenoids and polyketides |
| U1 vs U2 | up | 3 | Glycerolipid metabolism | 0.0582 | 0.1747 | Metabolism | Lipid metabolism |
| U1 vs U2 | up | 2 | Sphingolipid metabolism | 0.0575 | 0.1815 | Metabolism | Lipid metabolism |
| U1 vs U2 | up | 2 | Flavonoid biosynthesis | 0.0767 | 0.2000 | Metabolism | Biosynthesis of other secondary metabolites |
| U1 vs U2 | up | 2 | Autophagy - other | 0.0883 | 0.2118 | Cellular Processes | Transport and catabolism |
| U1 vs U2 | up | 2 | Alanine, aspartate and glutamate metabolism | 0.1003 | 0.2149 | Metabolism | Amino acid metabolism |
| U1 vs U2 | up | 1 | Glycosphingolipid biosynthesis - globo and isoglobo series | 0.0875 | 0.2188 | Metabolism | Glycan biosynthesis and metabolism |
| U1 vs U2 | up | 1 | Taurine and hypotaurine metabolism | 0.0954 | 0.2202 | Metabolism | Metabolism of other amino acids |
| U1 vs U2 | up | 1 | Monobactam biosynthesis | 0.0994 | 0.2208 | Metabolism | Biosynthesis of other secondary metabolites |
| U1 vs U2 | up | 2 | Arginine and proline metabolism | 0.1114 | 0.2304 | Metabolism | Amino acid metabolism |
| U1 vs U2 | up | 1 | Butanoate metabolism | 0.1747 | 0.3381 | Metabolism | Carbohydrate metabolism |
| U1 vs U2 | up | 3 | Ubiquitin mediated proteolysis | 0.1728 | 0.3456 | Genetic Information Processing | Folding, sorting and degradation |
| U1 vs U2 | up | 1 | Histidine metabolism | 0.1995 | 0.3741 | Metabolism | Amino acid metabolism |
| U1 vs U2 | up | 2 | Glycine, serine and threonine metabolism | 0.2194 | 0.3989 | Metabolism | Amino acid metabolism |
| U1 vs U2 | up | 2 | Phosphatidylinositol signaling system | 0.2435 | 0.4173 | Environmental Information Processing | Signal transduction |
| U1 vs U2 | up | 2 | Glutathione metabolism | 0.2370 | 0.4183 | Metabolism | Metabolism of other amino acids |
| U1 vs U2 | up | 2 | Pyruvate metabolism | 0.2724 | 0.4302 | Metabolism | Carbohydrate metabolism |
| U1 vs U2 | up | 1 | Other glycan degradation | 0.2665 | 0.4322 | Metabolism | Glycan biosynthesis and metabolism |
| U1 vs U2 | up | 1 | Sulfur metabolism | 0.2601 | 0.4334 | Metabolism | Energy metabolism |
| U1 vs U2 | up | 4 | Plant hormone signal transduction | 0.2832 | 0.4357 | Environmental Information Processing | Signal transduction |
| U1 vs U2 | up | 1 | Nitrogen metabolism | 0.2917 | 0.4376 | Metabolism | Energy metabolism |
| U1 vs U2 | up | 1 | Arginine biosynthesis | 0.3279 | 0.4799 | Metabolism | Amino acid metab |
| U1 vs U2 | down | 8 | Starch and sucrose metabolism | 0.0009 | 0.0418 | Metabolism | Carbohydrate metabolism |
| U1 vs U2 | down | 3 | Photosynthesis - antenna proteins | 0.0033 | 0.0763 | Metabolism | Energy metabolism |
| U1 vs U2 | down | 2 | Other types of O-glycan biosynthesis | 0.0094 | 0.1434 | Metabolism | Glycan biosynthesis and metabolism |
| U1 vs U2 | down | 1 | Biosynthesis of various secondary metabolites - part 2 | 0.0162 | 0.1487 | Metabolism | Biosynthesis of other secondary metabolites |
| U1 vs U2 | down | 6 | Phenylpropanoid biosynthesis | 0.0142 | 0.1628 | Metabolism | Biosynthesis of other secondary metabolites |
| U1 vs U2 | down | 1 | Mannose type O-glycan biosynthesis | 0.0321 | 0.2458 | Metabolism | Glycan biosynthesis and metabolism |
| U1 vs U2 | down | 2 | Zeatin biosynthesis | 0.0554 | 0.2547 | Metabolism | Metabolism of terpenoids and polyketides |
| U1 vs U2 | down | 7 | Plant hormone signal transduction | 0.0517 | 0.2643 | Environmental Information Processing | Signal transduction |
| U1 vs U2 | down | 4 | Glyoxylate and dicarboxylate metabolism | 0.0418 | 0.2746 | Metabolism | Carbohydrate metabolism |
| U1 vs U2 | down | 3 | Galactose metabolism | 0.0490 | 0.2818 | Metabolism | Carbohydrate metabolism |
| U1 vs U2 | down | 2 | Base excision repair | 0.1024 | 0.3140 | Genetic Information Processing | Replication and repair |
| U1 vs U2 | down | 3 | Glycerolipid metabolism | 0.0971 | 0.3438 | Metabolism | Lipid metabolism |
| U1 vs U2 | down | 3 | Glycine, serine and threonine metabolism | 0.0971 | 0.3438 | Metabolism | Amino acid metabolism |
| U1 vs U2 | down | 2 | Phenylalanine, tyrosine and tryptophan biosynthesis | 0.0940 | 0.3601 | Metabolism | Amino acid metabolism |
| U1 vs U2 | down | 3 | Carbon fixation in photosynthetic organisms | 0.0866 | 0.3620 | Metabolism | Energy metabolism |
| U1 vs U2 | down | 1 | Vitamin B6 metabolism | 0.1458 | 0.3726 | Metabolism | Metabolism of cofactors and vitamins |
| U1 vs U2 | down | 2 | Cyanoamino acid metabolism | 0.1435 | 0.3884 | Metabolism | Metabolism of other amino acids |
| U1 vs U2 | down | 2 | Porphyrin and chlorophyll metabolism | 0.1380 | 0.3967 | Metabolism | Metabolism of cofactors and vitamins |
| U1 vs U2 | down | 2 | Circadian rhythm - plant | 0.1662 | 0.4024 | Organismal Systems | Environmental adaptation |
