# Supplementary material for: Transcriptome comparison analyses in UV-B induced AsA accumulation of Lactuca sativa L
Source: BMC Genomics. 2023 Feb 3;24:61. doi: 10.1186/s12864-023-09133-7 (PMC9896689; doi:10.1186/s12864-023-09133-7)
Supplement: Supplementary file 4 — Additional file 4: Table S2. Unigenes involved in AsA biosynthesis and metabolism in lettuce. [file 12864_2023_9133_MOESM4_ESM.doc]

**Table S2** Unigenes involved in AsA biosynthesis and metabolism in lettuce

| **Unigene** | **Gene id** | **FPKM of C** | **FPKM of U1** | **FPKM of U2** | **Log_2_FC**  **(CvsU1)** | **Log_2_FC**  **(CvsU2)** | **Log_2_FC**  **(U1vsU2)** | ***P*-adjust (CvsU1)** | ***P*-adjust (CvsU2)** | ***P*-adjust (U1vsU2)** |
| --- | --- | --- | --- | --- | --- | --- | --- | --- | --- | --- |
| *MIOX* | LG4414077 | 2.54 | 1.79 | 15.25 | 0.50 | 2.59 | 3.09 | 0.77 | 0.00 | 0.00 |
| *MIOX* | LG8718858 | 12.40 | 14.10 | 54.40 | 0.19 | 2.13 | 1.95 | 0.86 | 0.00 | 0.00 |
| *GME* | LG3259813 | 288.67 | 247.75 | 318.53 | 0.22 | 0.14 | 0.36 | 0.59 | 0.08 | 0.00 |
| *GGP/ VTC2* | LG5505590 | 46.31 | 53.19 | 51.49 | 0.20 | 0.15 | 0.05 | 0.05 | 0.08 | 0.88 |
| *GGP/ VTC2* | LG7642189 | 99.37 | 122.91 | 105.35 | 0.31 | 0.08 | 0.22 | 0.00 | 0.40 | 0.41 |
| *GPP/ VTC4* | LG4334981 | 68.97 | 66.10 | 54.32 | 0.06 | 0.34 | 0.28 | 0.95 | 0.20 | 0.32 |
| *GaIDH* | LG9826032 | 84.90 | 82.32 | 83.36 | 0.04 | 0.03 | 0.02 | 0.76 | 0.55 | 0.97 |
| *GLDH* | LG5523038 | 21.26 | 18.73 | 18.65 | 0.18 | 0.19 | 0.01 | 0.75 | 0.59 | 1.00 |
| *NPP* | LG7629544 | 18.36 | 20.25 | 13.89 | 0.14 | 0.40 | 0.54 | 0.34 | 0.12 | 0.00 |
| *GaIUR* | LG8753218 | 5.67 | 5.06 | 2.30 | 0.16 | 1.30 | 1.14 | 0.85 | 0.00 | 0.00 |
| *GaIUR* | LG8753187 | 0.54 | 0.91 | 0.86 | 0.75 | 0.67 | 0.08 | 0.67 | 0.61 | 0.95 |
| *GaIUR* | LG3270461 | 0.14 | 0.39 | 0.48 | 1.48 | 1.78 | 0.30 | 1.00 | 0.28 | 1.00 |
| *APX* | LG8848427 | 355.30 | 326.20 | 207.24 | 0.12 | 0.78 | 0.65 | 0.94 | 0.00 | 0.00 |
| *APX* | LG8848770 | 0.92 | 0.48 | 1.06 | 0.94 | 0.20 | 1.14 | 1.00 | 0.88 | 1.00 |
| *APX* | LG3298085 | 0.41 | 0.24 | 0.21 | 0.77 | 0.97 | 0.19 | 0.80 | 0.65 | 1.00 |
| *APX* | LG6596493 | 128.01 | 122.52 | 118.13 | 0.06 | 0.12 | 0.05 | 0.98 | 0.96 | 0.94 |
| *APX* | LG3269273 | 6.55 | 4.23 | 3.40 | 0.63 | 0.95 | 0.32 | 0.31 | 0.12 | 0.74 |
| *APX* | LG6542460 | 12.57 | 8.78 | 8.93 | 0.52 | 0.49 | 0.02 | 0.10 | 0.07 | 0.96 |
| *APX* | LG3301259 | 364.16 | 372.79 | 370.68 | 0.03 | 0.03 | 0.01 | 0.49 | 0.47 | 0.99 |
| *APX* | LG1133571 | 148.26 | 151.98 | 154.88 | 0.04 | 0.06 | 0.03 | 0.64 | 0.24 | 0.91 |
| *AO* | LG7605270 | 8.83 | 4.04 | 3.95 | 1.13 | 1.16 | 0.03 | 0.00 | 0.02 | 0.98 |
| *AO* | LG8726916 | 4.09 | 1.32 | 1.22 | 1.63 | 1.75 | 0.11 | 0.00 | 0.00 | 0.91 |
| *AO* | LG2179079 | 0.34 | 0.31 | 0.25 | 0.13 | 0.44 | 0.31 | 0.98 | 0.79 | 0.85 |
| *AO* | LG4332186 | 19.29 | 12.63 | 13.54 | 0.61 | 0.51 | 0.10 | 0.01 | 0.40 | 0.90 |
| *AO* | LG4396216 | 11.29 | 16.36 | 11.66 | 0.54 | 0.05 | 0.49 | 0.15 | 0.80 | 0.24 |
| *AO* | LG4361023 | 1.05 | 0.91 | 1.00 | 0.21 | 0.07 | 0.14 | 0.95 | 0.99 | 0.92 |
| *AO* | LG9765162 | 5.58 | 8.44 | 6.17 | 0.60 | 0.15 | 0.45 | 0.01 | 0.69 | 0.46 |
| *AO* | LG7666613 | 7.04 | 6.80 | 6.91 | 0.50 | 2.59 | 3.09 | 0.96 | 0.85 | 0.96 |
| *AO* | LG9801575 | 86.63 | 44.35 | 43.71 | 0.19 | 2.13 | 1.95 | 0.00 | 0.28 | 0.99 |
| *AO* | G7650844 | 22.34 | 15.78 | 11.18 | 0.22 | 0.14 | 0.36 | 0.13 | 0.00 | 0.19 |
| *AO* | LG4336085 | 1.39 | 1.05 | 0.97 | 0.20 | 0.15 | 0.05 | 0.74 | 0.59 | 1.00 |
| *AO* | LG8674826 | 2.29 | 1.55 | 0.87 | 0.31 | 0.08 | 0.22 | 0.44 | 0.02 | 0.28 |
| *MDHAR* | LG2229156 | 214.33 | 323.42 | 361.65 | 0.06 | 0.34 | 0.28 | 0.00 | 0.00 | 0.59 |
| *MDHAR* | LG0112562 | 4.03 | 2.86 | 2.65 | 0.04 | 0.03 | 0.02 | 0.36 | 0.13 | 0.87 |
| *MDHAR* | LG6584635 | 50.56 | 43.46 | 24.34 | 0.18 | 0.19 | 0.01 | 0.62 | 0.00 | 0.00 |
| *MDHAR* | LG6577824 | 131.67 | 126.20 | 128.43 | 0.14 | 0.40 | 0.54 | 0.91 | 0.61 | 0.87 |
| *DHAR* | LG2216326 | 46.97 | 44.11 | 32.46 | 0.16 | 1.30 | 1.14 | 0.97 | 0.00 | 0.01 |
| *DHAR* | LG8760079 | 25.99 | 26.35 | 23.76 | 0.75 | 0.67 | 0.08 | 0.67 | 0.92 | 0.51 |
| *GR* | LG9818853 | 60.96 | 65.12 | 56.50 | 1.48 | 1.78 | 0.30 | 0.21 | 0.92 | 0.18 |
| *GR* | LG196915 | 97.76 | 79.21 | 111.96 | 0.12 | 0.78 | 0.65 | 0.13 | 0.04 | 0.00 |
| *GST* | LG5497179 | 68.92 | 32.86 | 31.42 | 0.94 | 0.20 | 1.14 | 0.00 | 0.00 | 0.90 |
| *GST* | LG3262677 | 1.78 | 7.15 | 185.50 | 0.77 | 0.97 | 0.19 | 0.00 | 0.00 | 0.01 |
| *GST* | LG7668694 | 245.66 | 228.64 | 255.55 | 0.06 | 0.12 | 0.05 | 0.98 | 0.29 | 0.48 |
| *GGT1_5* | LG5479175 | 1.80 | 3.34 | 10.26 | 0.63 | 0.95 | 0.32 | 0.01 | 0.00 | 0.00 |
| *GGT1_5* | LG7640547 | 29.41 | 36.64 | 50.73 | 0.52 | 0.49 | 0.02 | 0.01 | 0.00 | 0.11 |
| *RRM2* | LG2244562 | 3.57 | 1.70 | 1.52 | 0.03 | 0.03 | 0.01 | 0.03 | 0.01 | 0.86 |
| *lacZ* | LG2195371 | 33.15 | 41.87 | 84.11 | 0.04 | 0.06 | 0.03 | 0.23 | 0.00 | 0.00 |
| *ALDH 2* | LG2189009 | 70.87 | 86.66 | 165.65 | 1.13 | 1.16 | 0.03 | 0.25 | 0.00 | 0.00 |
| *ALDH 7* | LG5515138 | 82.11 | 117.45 | 369.85 | 1.63 | 1.75 | 0.11 | 0.58 | 0.00 | 0.02 |
| *RAFS* | LG5484313 | 62.09 | 58.64 | 224.82 | 0.13 | 0.44 | 0.31 | 1.00 | 0.00 | 0.00 |
| *RAFS* | LG5485269 | 41.19 | 77.40 | 142.19 | 0.61 | 0.51 | 0.10 | 0.81 | 0.00 | 0.01 |
| *gaIA* | LG2210016 | 46.37 | 48.94 | 157.94 | 0.54 | 0.05 | 0.49 | 0.75 | 0.00 | 0.00 |
| *galE* | LG6581247 | 10.44 | 12.51 | 28.46 | 0.21 | 0.07 | 0.14 | 0.64 | 0.00 | 0.02 |
| *AOX1* | LG5512390 | 10.37 | 11.53 | 16.17 | 0.60 | 0.15 | 0.45 | 0.42 | 0.00 | 0.01 |
| *AOX1* | LG5492871 | 51.14 | 143.79 | 168.57 | 0.50 | 2.59 | 3.09 | 0.00 | 0.00 | 0.73 |
| *AOX1* | LG7668143 | 34.83 | 41.29 | 52.55 | 0.19 | 2.13 | 1.95 | 0.44 | 0.00 | 0.07 |
| *petB* | LG9796785 | 5.70 | 6.50 | 9.12 | 0.22 | 0.14 | 0.36 | 0.57 | 0.02 | 0.05 |
| *petA* | LG1148423 | 27.34 | 35.43 | 59.76 | 0.20 | 0.15 | 0.05 | 0.01 | 0.00 | 0.01 |
| *petA* | LG3289553 | 33.66 | 49.02 | 73.32 | 0.31 | 0.08 | 0.22 | 0.00 | 0.00 | 0.12 |
| *petA* | LG7605437 | 27.34 | 35.43 | 59.76 | 0.06 | 0.34 | 0.28 | 0.01 | 0.00 | 0.01 |
| *atpB* | LG2174170 | 0.94 | 2.24 | 2.80 | 0.04 | 0.03 | 0.02 | 0.17 | 0.01 | 0.78 |
