# Supplementary material for: Transcriptome comparison analyses in UV-B induced AsA accumulation of Lactuca sativa L
Source: BMC Genomics. 2023 Feb 3;24:61. doi: 10.1186/s12864-023-09133-7 (PMC9896689; doi:10.1186/s12864-023-09133-7)
Supplement: Supplementary file 5 — Additional file 5: Table S3. Transcript levels of UVR8 and HY5 in the three treatments. [file 12864_2023_9133_MOESM5_ESM.doc]

**Table S3 Transcript levels of UVR8 and HY5 in the three treatments**

| **Unigene** | **C FPKM** | **U1 FPKM** | **U2 FPKM** | **Log_2_FC (CvsU1)** | **Log_2_FC**  **(CvsU2)** | **Log_2_FC**  **(U1vsU2)** | ***P-*adjust**  **(C vs U1)** | 1. **adjust**   **(CvsU2)** | ***P-*adjust (U1vsU2)** |
| --- | --- | --- | --- | --- | --- | --- | --- | --- | --- |
| *UVR8*(LG3280295) | 8.25 | 8.92 | 8.83 | 0.11 | 0.10 | 0.01 | 0.46 | 0.52 | 0.92 |
| *UVR8*(LG7632820) | 22.99 | 23.20 | 24.82 | 0.01 | 0.11 | 0.10 | 0.71 | 0.16 | 0.56 |
| *UVR8*(LG5498155) | 44.35 | 51.83 | 41.06 | 0.22 | 0.11 | 0.34 | 0.02 | 0.94 | 0.04 |
| *UVR8*(LG2221258) | 20.69 | 22.25 | 26.59 | 0.10 | 0.36 | 0.26 | 0.36 | 0.00 | 0.12 |
| *UVR8*(LG2200221) | 31.12 | 35.76 | 49.02 | 0.20 | 0.66 | 0.46 | 0.15 | 0.00 | 0.03 |
| *UVR8*(LG5433853) | 17.39 | 21.33 | 16.46 | 0.29 | 0.08 | 0.37 | 0.01 | 0.88 | 0.09 |
| *UVR8*(LG1145107) | 13.17 | 15.35 | 17.41 | 0.22 | 0.40 | 0.18 | 0.21 | 0.01 | 0.56 |
| *UVR8*(LG5500228) | 11.24 | 11.99 | 11.65 | 0.09 | 0.05 | 0.04 | 0.55 | 0.51 | 0.92 |
| *HY5*(LG7601578) | 13.45 | 14.84 | 16.34 | 0.14 | 0.28 | 0.14 | 0.13 | 0.00 | 0.40 |
| *HY5*(LG5453544) | 14.17 | 8.56 | 10.48 | 0.73 | 0.44 | 0.29 | 0.04 | 0.33 | 0.52 |
| *HY5*(LG6556652) | 3.68 | 2.39 | 4.45 | 0.62 | 0.27 | 0.90 | 0.63 | 0.51 | 0.16 |
