# Supplementary material for: Transcriptome comparison analyses in UV-B induced AsA accumulation of Lactuca sativa L
Source: BMC Genomics. 2023 Feb 3;24:61. doi: 10.1186/s12864-023-09133-7 (PMC9896689; doi:10.1186/s12864-023-09133-7)
Supplement: Supplementary file 6 — Additional file 6: Table S4. Primers for real time RT-PCR analysis. [file 12864_2023_9133_MOESM6_ESM.doc]

**Table S4 Primers for real time RT-PCR analysis**

| **Unigene** | **Forward primer (5’-3’)** | **Reverse primer (5’-3’)** |
| --- | --- | --- |
| *galE*(LG6581247) | GCACATACAATCTCACTTGC | TGACTCCCTATATATCCGGC |
| *MIOX*(LG8718858) | AGATGGAGGGTTTGTAGTGC | AATTCTTCCACCTGCTTCTG |
| *DHAR*(LG2216326) | AGGAGAAGCATCTTCCTTAC | AGTCAACAATGGTGGTTCAG |
| *ALDH*(LG2189009) | TAGACTGAAAACAGGAGTCG | ATGAGGAGCTTGGTGTATTC |
| *ALDH*(LG2189009) | TAGACTGAAAACAGGAGTCG | TATTCAACCTTGACAGGTGG |
| *lacZ*(LG2195371) | TGCTCAAAGTCCTACCACTG | GGGTGGATCAAGTGGAAATG |
| *NADH*(LG4385076) | AATCAGTCGATTCTCTACAG | GATTCTCCCATTTCTGTGTC |
| *APX*(LG3301259) | TACGATGTGAACACCAAGAC | GTAACCTCCACTGCAACAAC |
| *APX*(LG8848427） | GCCCTGATGTTCCCTTCCAT | TTGTGTGCAGCTCCAAGAGT |
| *AO*(LG7605270) | CACGAATTGCCCCATTCCAC | TGGCCTCCGTCTAGAATTGC |
| *MDHAR*(LG2229156) | AGTTTCGGTGCGTATTGGGT | CCACGGATACACCGCTTTCT |
| *GLA*(LG2210016) | ATGCCGGATCTCTAACATGC | ATAAAAAATGGGCCTTCCTG |
| *GME*(LG3259813） | CCACATACCAGGCCCAGAAG | GCAGCCCTAAGAGAACCGAG |
| *GGP*(LG5505590） | CCTCCCTCAGTGTTATGCGG | TCACCCAAACTTCCAGCCTC |
| *GGP2*(LG7642189） | ACTACCAGAAGGAGGGGGTC | GCCACAAAACCGAGTTCACC |
| *GR*(LG196915） | TGTGGCGATGAAGTGTGGAG | CAGCAATCCTCCGTGTGACT |
| *GAIUR*(LG8753218） | AGGGAAGACAGTGGCTCAGA | ATGCCTGCGTTGAGGAATCT |
| *GAIDH*(LG9826032） | ACGAGACAATCCCTGCACTC | CTGGATGCCAATCTGGAGGG |
| *GAPDH*(LG4384339) | GATCAAGATCGGAATCAACG | AAAAGAAGGGTTTTGTCGTC |
